# Immunostimulatory Defective Viral Genomes from Respiratory Syncytial Virus Promote a Strong Innate Antiviral Response during Infection in Mice and Humans

**DOI:** 10.1371/journal.ppat.1005122

**Published:** 2015-09-03

**Authors:** Yan Sun, Deepika Jain, Cynthia J. Koziol-White, Emmanuelle Genoyer, Micah Gilbert, Karla Tapia, Reynold A. Panettieri, Richard L. Hodinka, Carolina B. López

**Affiliations:** 1 Department of Pathobiology, School of Veterinary Medicine, University of Pennsylvania, Philadelphia, Pennsylvania, United States of America; 2 Department of Medicine, Perelman School of Medicine at the University of Pennsylvania, Philadelphia, Pennsylvania, United States of America; 3 Department of Pathology and Laboratory of Medicine, Perelman School of Medicine at the University of Pennsylvania and Clinical Virology Laboratory, Children’s Hospital of Philadelphia, Philadelphia, Pennsylvania, United States of America; St. Jude Children's Research Hospital, UNITED STATES

## Abstract

Human respiratory syncytial virus (RSV) is a major cause of severe respiratory illness in children and susceptible adults. RSV blocks the development of the innate antiviral immune response and can grow to high titers in the respiratory tract. Here we demonstrate that immunostimulatory defective viral genomes (iDVGs) that are naturally generated during RSV replication are strong inducers of the innate antiviral response to RSV in mice and humans. In mice, RSV iDVGs stimulated the expression of antiviral genes, restricted viral replication, and prevented weight loss and lung inflammation. In human cells, the antiviral response to RSV iDVGs was dominated by the expression of IFN-λ1 over IFN-β and was driven by rapid intranuclear accumulation of the transcription factor IRF1. RSV iDVGs were detected in respiratory secretions of hospitalized patients, and their amount positively correlated with the level of expression of antiviral genes in the samples. Infection of explanted human lung tissue from different donors revealed that most humans can respond to RSV iDVGs and that the rate of accumulation of iDVGs during infection directly correlates with the quality of the antiviral response. Taken together, our data establish iDVGs as primary triggers of robust antiviral responses to RSV and provide the first evidence for an important biological role for naturally occurring iDVGs during a *paramyxovirus* infection in humans.

## Author Summary

Respiratory syncytial virus is a major cause of chronic lung damage, asthma exacerbations, and hospitalizations of infants, elders, and high-risk adults. Currently, there is no effective vaccine or treatment available to protect the general population from RSV infection. Here, we demonstrate that defective forms of RSV genomes naturally generated during infection effectively stimulate the antiviral response *in vitro* and *in vivo*. In human cells, RSV iDVGs trigger the antiviral response through a mechanism characterized by the potent activation of the transcription factor IRF1 and a dominant expression of the type III IFN gene IFNL1 (IFN-λ1). This study establishes for the first time that naturally occurring iDVGs trigger robust host antiviral responses to RSV in mice and humans and reveals new opportunities to potentiate the host response to RSV infection and minimize viral-induced pathology.

## Introduction

According to current paradigms, the host immune response to viral infection is initiated upon recognition of molecular motifs generally present as part of the viral genome (reviewed in [1–3]). However, most viruses of clinical significance interfere with the host innate immune response allowing the virus to replicate to high titers before being controlled by the immune system [4–6]. The failure to respond to actively replicating virus reveals a fundamental paradox about the identity of viral elements key to initiating immune recognition during natural infections.

During peak replication *in vitro*, *paramyxoviruses*, including parainfluenza virus, measles, and the respiratory syncytial virus (RSV), produce defective viral genomes (DVGs) that contain large genomic deletions and are unable to replicate in the absence of helper virus [7–11]. Viruses with a high content of the “copy-back” type of DVGs can strongly stimulate the expression of antiviral genes in infected cells in culture [12–15] and in mice [16, 17] by stimulating a signaling cascade initiated by the intracellular pathogen recognition receptors retinoic acid-inducible gene 1 (RIG-I) and melanoma differentiation-associated protein 5 (MDA5) [17–19]. Our recent studies in mice infected with the murine parainfluenza virus Sendai (SeV) suggest that immunostimulatory DVGs (iDVGs) that trigger the signaling of RIG-I like receptors (RLRs) accumulate *in situ* during experimental infection, thereby promoting virus clearance and reducing virulence [17]. These findings strongly argue against the traditional view of DVG accumulation as an artifact of *in vitro* viral replication. Despite of descriptive studies of DVGs in patients infected with a growing number of viruses [20–23], it is unknown whether iDVGs arise during natural *paramyxovirus* infections in humans or if they impact the host response to infection.

Respiratory Syncytial Virus (RSV) is the most common cause of bronchiolitis and pneumonia among infants [24, 25] and severe infant RSV bronchiolitis is an important cause of the development of asthma later in life [25–29]. RSV infects all humans before two years of age and in most patients causes a mild cold-like disease. Currently, there is no means of predicting the clinical outcome of infection, nor vaccines or therapeutic strategies to protect the general population. The RSV-encoded proteins NS1 and NS2 potently block the innate host response to infection, permitting the virus to replicate to high titers and promoting pathology [30–32]. Clearance of the virus in these conditions depends on the cellular immune response, which worsens lung pathology [33–36]. Elucidation of mechanisms that overcome viral immune response antagonists leading to the control of viral replication independent of cellular immunity will reveal potentially new strategies to minimize post-viral lung disease. DVGs have been described in RSV passaged in cell cultures at high titers [9], but it is unknown whether they arise during natural RSV infections in humans or whether they reduce viral replication and pathogenesis.

This manuscript describes our investigation of the role of iDVGs during RSV infection. We demonstrate for the first time that iDVGs accumulate naturally during *paramyxovirus* infection in humans and that they are critical triggers of the innate antiviral response during RSV infection.

## Results

### RSV iDVGs prevent lung pathology in mice

**Fig 1:**
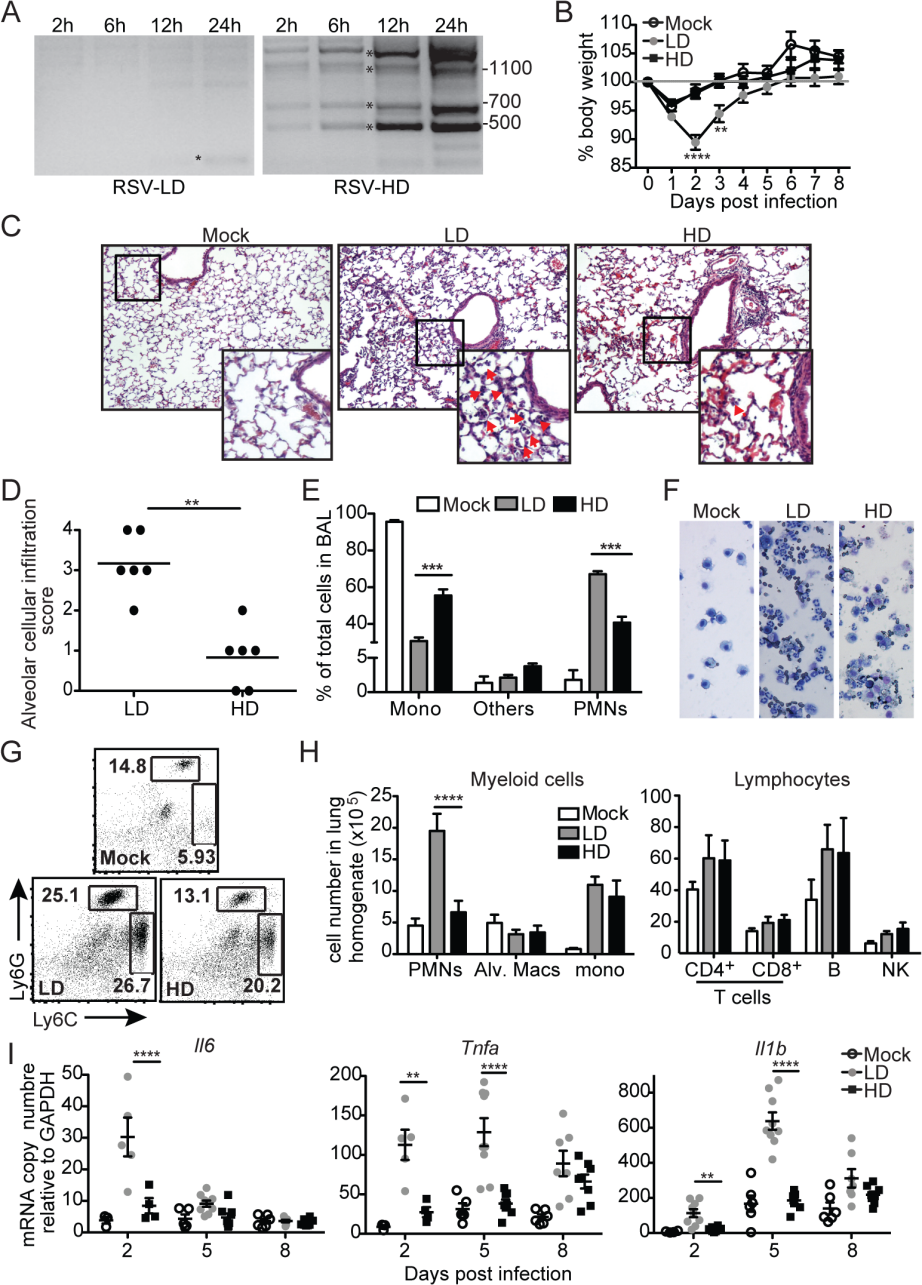
RSV DVGs prevent viral pathogenesis in vivo. (A) Hep2 cells were infected with RSV-LD or HD at a moi of 1.5 TCID_50_/cell and DVGs were detected by PCR at the indicated times. Details of the PCR assay can be seen in S1 Fig and sequences of the amplicons labeled with a star can be found in S2 Fig Base pair size references are indicated in the gel. (B) Mice weight loss was monitored overtime. Error bars indicate standard deviation of data pooled from two independent experiments (n = 7–9 mice per group total; **p<0.01, ****p<0.0001 by two-way ANOVA with Bonferroni post hoc test. Variance was not significantly different between groups as per Bartlett’s test). (C) Representative H&E staining for lung sections from mock, RSV-LD, or RSV-HD-infected mice on day 2 post infection. Picture magnification: 10X; insert is a digital amplification. Red arrows indicate alveolar cellular infiltrate. (D) Pathology score for alveolar infiltration in the lung (n = 6 mice per group; **p<0.01, by two-tailed Mann Whitney test). (E) Differential counts from cytospins from mice bronchoalveolar lavage (BAL) on day 2 post infection (Mono: monocytes and macrophages, PMNs: polymorphonuclear cells; ***p<0.001 by two-way ANOVA with Bonferroni’s post hoc test, n = 5–8 mice per group). (F) Representative cytospin images (20X). (G) Representative flow cytometry plots from whole lung single cell suspensions on day 2 post infection. Plots are pre-gated in singlets, live, CD45^+^CD11b^+^ cells. (H) Quantification of different cell types in the lung of infected mice on day 2 post infection (****p<0.0001 by two-way ANOVA with Bonferroni’s post hoc test, n = 5–8 mice per group, Alv. Macs: alveolar macrophages). (I) Expression of pro-inflammatory genes in whole lung tissue on day 2, 5, and 8 post infection. (n = 3–5 mice per group, *p<0.05, **p<0.01 by one-way ANOVA with Bonferroni’s post hoc test).

To investigate the impact of RSV DVGs on lung pathology upon infection *in vivo*, BALB/c mice were infected with identical infectious doses of RSV stocks depleted of DVGs (RSV-LD) or with a high content of DVGs (RSV-HD). In control experiments, we confirmed that RSV-LD failed to accumulate significant amounts of copy-back DVGs in infected cells until 24 h post infection, while cells infected with RSV-HD had high levels of various species of copy-back DVGs from early time points (Fig 1A; detailed PCR strategy on S1 Fig and sequences of labeled products on S2 Fig). In mice, infection in the absence of DVGs (RSV-LD) resulted in more pronounced weight loss (Fig 1B) accompanied by a significantly enhanced alveolar cellular infiltrate (Fig 1C and 1D). No significant differences were observed at the level of peribronchial and perivascular inflammation in the lungs (S3 Fig). In mice infected with RSV-LD, the cellular infiltrate at the time of peak weight loss (day 2 post infection) was enriched in neutrophils over monocytes and macrophages compared with the infiltrate in mice infected with RSV-HD (Fig 1E–1H; gating in S4 Fig). No differences in the composition of pulmonary lymphocytes (T, B, or NK cells) were observed between infections with RSV-LD and HD at this time point (Fig 1H). The enhanced myeloid cellular infiltrate in the lungs of mice infected with RSV-LD associated with increased expression of a number of pro-inflammatory genes including *Il6*, *Tnf*, *and Il1b* (Fig 1I). It is noted that *Tnf* and *Il1b* mRNA expression were sustained at D5 post RSV-LD infection, while *Il6* mRNA was only induced acutely at early times of infection, corresponding with the peak of weight loss. Overall, these data demonstrate that a high content of iDVGs in RSV stocks prevents excessive inflammation and accumulation of neutrophils before evidence of significant T cell recruitment into the lung.

### RSV iDVGs strongly stimulate the innate antiviral response

**Fig 2:**
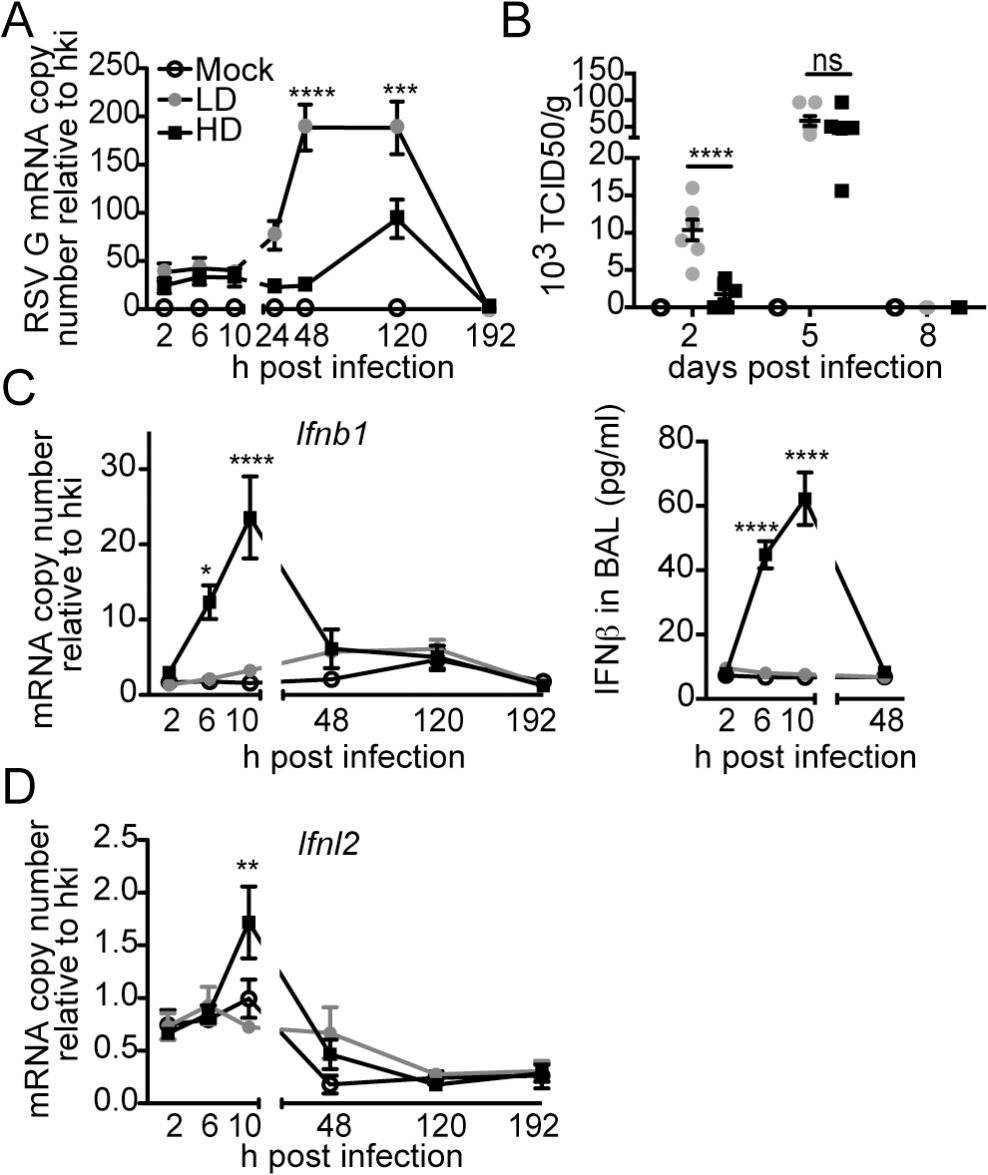
RSV iDVGs rapidly induce an antiviral response that controls viral load in mice. Balb/c mice were infected intranasally with 5 x 10^6^ TCID_50_/mouse of RSV-LD or HD, or mock infected. Expression of (A) RSV G mRNA and (B) virus titer in whole lung homogenates at the indicated time points. (C) Ifnb1 mRNA and IFNβ protein in the bronchoalveolar lavage (BAL). (D) Expression of Ifnl2 in whole lung homogenates. Error bars indicate mean ± SEM of individual measurements in two pooled independent experiments each with triplicate technical measurements. (n = 7–9 mice per group total; *p<0.05, **p<0.01, ***p<0.001, ****p<0.0001 by two-way ANOVA with Bonferroni’s post hoc test). Gene expression is shown as copy number relative to a house keeping gene expression index determined from the expression of Rps11 and α-tubulin.

To determine whether protection during infection with RSV-HD was due to an enhanced antiviral response in the lung compared to infection with RSV-LD, we measured viral titers and the expression of type I and III IFN genes through the course of infection. Infection with RSV-HD resulted in decreased viral mRNA and infectious titers in the lungs on day 2 post infection (Fig 2A and 2B) that associated with strong expression of IFNβ (Fig 2C) and *Ifnl2* (Fig 2D) detectable as early as 6 h post infection. In contrast, antiviral genes were not significantly expressed at any time point after infection with RSV-LD. Notably, equivalent amounts of infectious viruses were present in the lungs on day 5 post infection with RSV-LD and HD, indicating that while iDVGs delay virus growth, the standard virus eventually emerges. Interestingly, the virus that emerged on day 5 was unable to trigger detectable IFN responses (Fig 2C and 2D), agreeing with long standing evidence of intercalating “waves” of full-length and defective viral genomes during infection [37] (i.e. too many defective genomes interfere with viral replication reducing the full-length virus to almost negligible levels which, in turn, eliminates defective genomes that cannot replicate in the absence of viral proteins and allow the standard virus to re-emerge). To determine whether the reduced virulence of RSV-HD was influenced by type I IFN-independent interference with viral replication by DVGs, we examined the infectivity of RSV-LD and RSV-HD in the IFN-deficient Vero cells. In this system, the expression levels of RSV G mRNA and viral titers were identical in infections with LD and HD for up to 24 h (S5 Fig), indicating that the infectious virus contained in LD and HD stocks have similar potential to replicate and to produce new viral particles at early times post infection. Overall, these data suggest that RSV iDVGs protect from pathology by stimulating a fast antiviral response that effectively delays viral replication long enough for adaptive immunity to develop, thereby minimizing overall lung inflammation.

**Fig 3:**
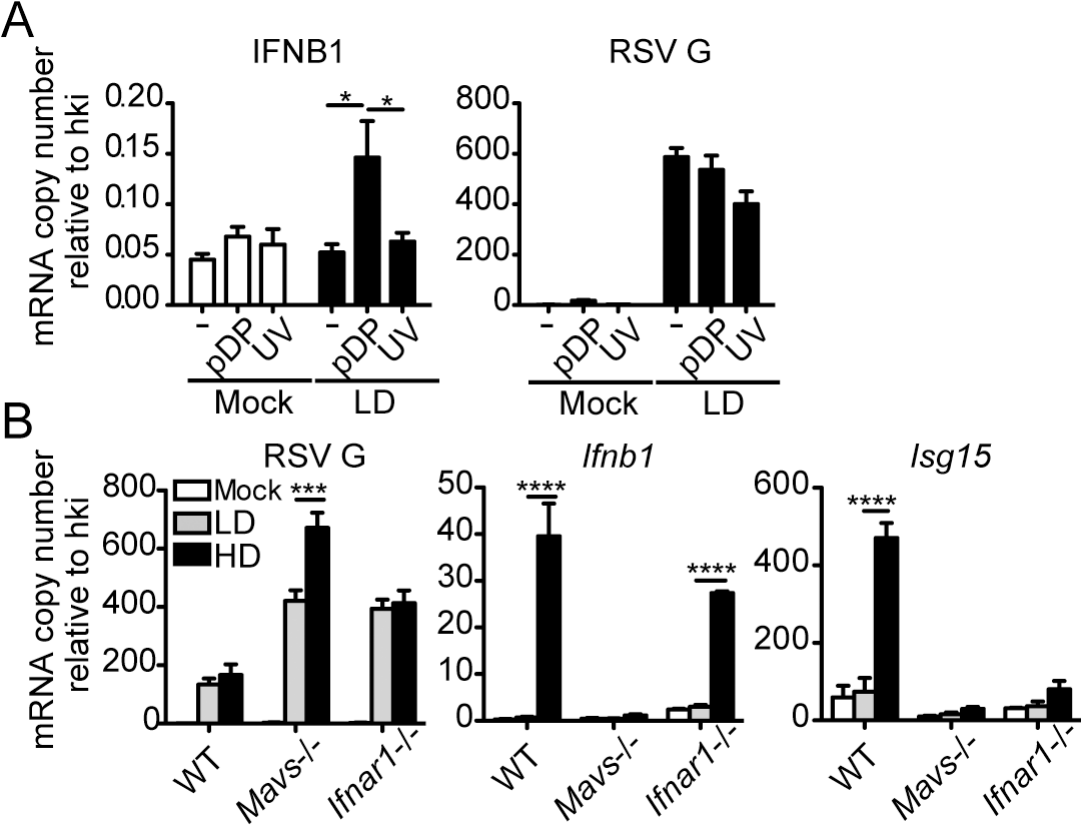
RSV iDVGs promote a MAVS-mediated antiviral response during RSV infection. (A) Expression of RSV G and antiviral genes in HEp2 cells infected for 10 h with RSV-LD at a moi of 1 TCID_50_/cell alone or in the presence of 20 pg/cell of purified defective particles (pDP) or UV-inactivated pDPs (UV). (B) Expression of RSV G and antiviral genes in wild type, Mavs^-/-^, or Ifnar1^-/-^ MEFs infected with RSV-LD or HD for 10 h at a moi of 1 TCID_50_/cell. Gene expression is shown as copy number relative to a house keeping gene expression index (hki) determined from the expression of ACTB (β-actin) and GAPDH (A) or Rps11 and α-tubulin (B). Error bars indicate mean ± SEM of three independent experiments each with triplicate technical measurements (*p<0.05, **p<0.01, ***p<0.001, ****p<0.0001 by two-way ANOVA with Bonferroni post hoc test).

To more directly assess the role of RSV iDVGs in the triggering of the antiviral response, defective viral particles containing iDVGs were purified from RSV-HD stocks (pDPs) and used to supplement *in vitro* infections with RSV-LD. Supplementation with pDPs promoted the expression of IFNB1 mRNA in the RSV-permissive human HEp-2 cells (Fig 3A) while inactivated UV-crosslinked pDPs failed to trigger the antiviral response, confirming the potent stimulatory ability of RSV iDVGs. The stimulatory activity of RSV-HD was lost in mouse embryo fibroblasts (MEFs) deficient in the adaptor protein MAVS (*Mavs* ^*-/-*^, Fig 3B), agreeing with the reported role of the intracellular viral sensors RIG-I and MDA5 in the recognition of *paramyxovirus* DVGs [18, 19, 38]. In contrast, the ability of RSV-HD to stimulate *Ifnb1* expression was largely maintained in cells deficient in the type I IFN receptor (*Ifnar1* ^*-/-*^, Fig 3B), while the control type I IFN-stimulated gene *Isf15* was only expressed in wild type cells. These data indicate that RSV iDVGs induce strong *Ifnb1* expression independent of type I IFN feedback. Notably, although both RSV-LD and RSV-HD replicated better in both *Mavs* ^-/-^ and *Ifnar1* ^-/-^ MEFs compared to wild type MEFs (Fig 3B), RSV-LD replicated to lower levels than RSV-HD in *Mavs* ^-/-^ cells suggesting that MAVS is involved in restricting viral replication in the presence of DVGs.

### RSV iDVGs stimulate an IRF1/IFNL1 axis in human cells

**Fig 4:**
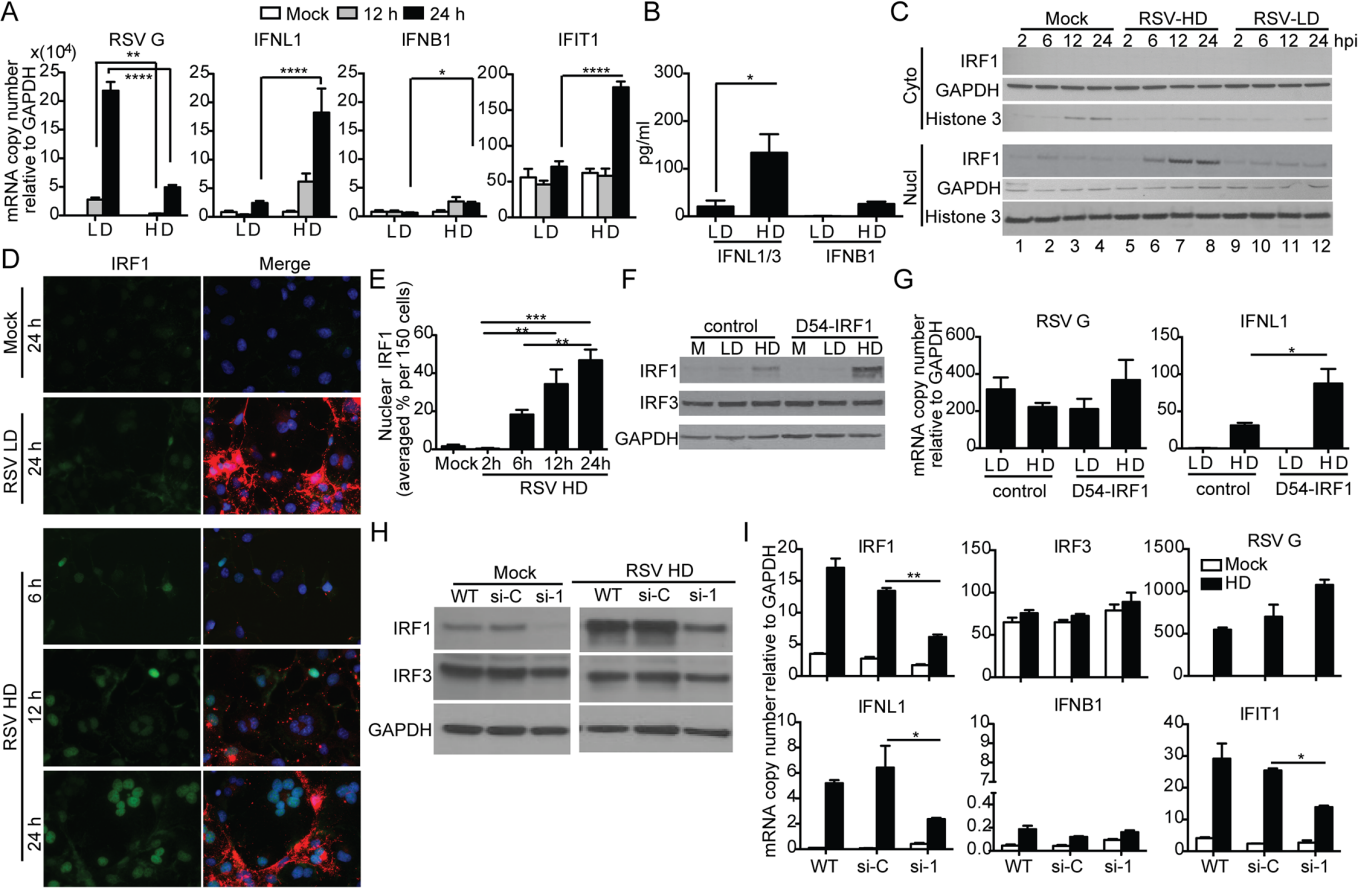
RSV iDVGs stimulate an IRF1/IFNL1-mediated antiviral response. A549 cells were infected with RSV-LD or RSV-HD at a moi of 1.5 TCID_50_/cell. Expression of (A) RSV G and antiviral genes mRNA, and (B) protein level of IFNB1 and IFNL1/3 in the cultures supernatants at 24 h post infection (*p<0.05 by one way unpaired t-test). (C, D) Cells were lysed or fixed at 2, 6, 12, and 24 h post infection for western blot (WB) and IFA. (C) For WB, nuclear and cytosolic fractions were immunoblotted for IRF1, GAPDH, and Histone 3. (D) For IFA, cells were co-stained for IRF1 (green, left panel), RSV F + G proteins (red in merged panel), and nuclei (blue). (E) Quantification of nuclear IRF1 upon iDVGs stimulation at designated time points post RSV-HD infection based on IFA images. (F, G) D54 control cells and D54 cells overexpressing IRF1 (D54-IRF1) were infected with RSV-HD at a moi of 1.5 TCID_50_/cell for 6 h. (F) IRF1 and IRF3 protein detected by WB from whole cell lysates. (G) Expression of RSV G and IFNL1 mRNA. (H, I) A549 cells were mock transfected (WT) or transfected with control siRNA (si-C), or IRF1 siRNA (si-1). After 40 h, the cells were mock infected or infected with RSV-HD at moi of 1.5 TCID_50_/cell. (H) WB for IRF3 and IRF1 was performed to confirm specific knockdown of IRF1 protein. (I) Expression of RSV G and other antiviral genes at 10 h post infection. Gene expression is shown as copy number relative to a house keeping gene expression index determined from the expression of ACTB (β-actin) and GAPDH. All error bars indicate mean ± SEM of at least three independent experiments (*p<0.05, **p<0.01, ***p<0.001, ****p<0.0001 by two-way ANOVA with Bonferroni post hoc test).

Infection of the human lung epithelial cell line A549 with RSV-HD resulted in the expression of IFNL1 (also known as IL-29), IFIT1 (also known as ISG56) and IFNB1; no expression of these genes was detected upon infection with RSV-LD, despite higher levels of viral replication (RSV G expression) (Fig 4A). Protein levels of IFNL1/3 and IFNB1 in the supernatants were consistent with mRNA expression (Fig 4B). Interestingly, expression of the type III IFN gene IFNL1 [39] was induced at much higher levels than IFNB1 in response to RSV-HD, indicating that RSV iDVGs predominantly induce an IFNL1-mediated antiviral response in A549 cells. Accordingly, the transcription factor IRF1, reported to be essential for MAVS-mediated IFNL1 expression [40–42], was detected in nuclear extracts as early as 6 h post infection with RSV-HD while it remained at basal levels in cells infected with RSV-LD or mock infected (Fig 4C). Accumulation of intranuclear IRF1 was confirmed by immunofluorescence (Fig 4D and 4E) and corresponded with enhanced IFNL1 mRNA expression (S6 Fig). Confirming a role for IRF1 as a mediator of the antiviral response induced by RSV iDVGs, cells overexpressing IRF1 (D54-IRF1; Fig 4F) showed significantly higher IFNL1 mRNA expression than control cells in response to RSV-HD, with no obvious differences in viral replication (Fig 4G). In addition, knockdown of IRF1 using siRNA (si-1; Fig 4H) significantly interfered with IFNL1 and IFIT1 mRNA expression in response to RSV-HD (Fig 4I). Notably, IRF1 knockdown was incomplete and the reduced amount of IRF1 protein observed upon infection with RSV-HD infected si-1 treated cells (Fig 4H) likely explains the low level of expression of IFNL1 observed upon infection of the knockdown cells. As control, cells treated with a non-targeting siRNA control (si-C) showed normal expression of IRF1, IFNL1, and IFIT1 mRNAs (Fig 4I). IRF1 siRNA neither impacted the expression of IRF3 before or after RSV-HD infection nor impaired the basal expression of antiviral genes, confirming the specificity of the IRF1 knockdown and negligible off-target effects (Fig 4I). Interestingly, both mRNA expression and protein level of IRF1 were significantly reduced in IRF3-knockdown cells (si-3) compared to the control cells (S7 Fig), suggesting that IRF3 is required for IRF1 expression in this system. Taken together, these observations indicate that an IRF3/IRF1/IFNL1 pathway is a critical component of the antiviral response induced by RSV iDVGs in human lung epithelial cells.

### Detection of iDVGs associate with expression of antiviral genes in respiratory secretions from pediatric patients infected with RSV

**Fig 5:**
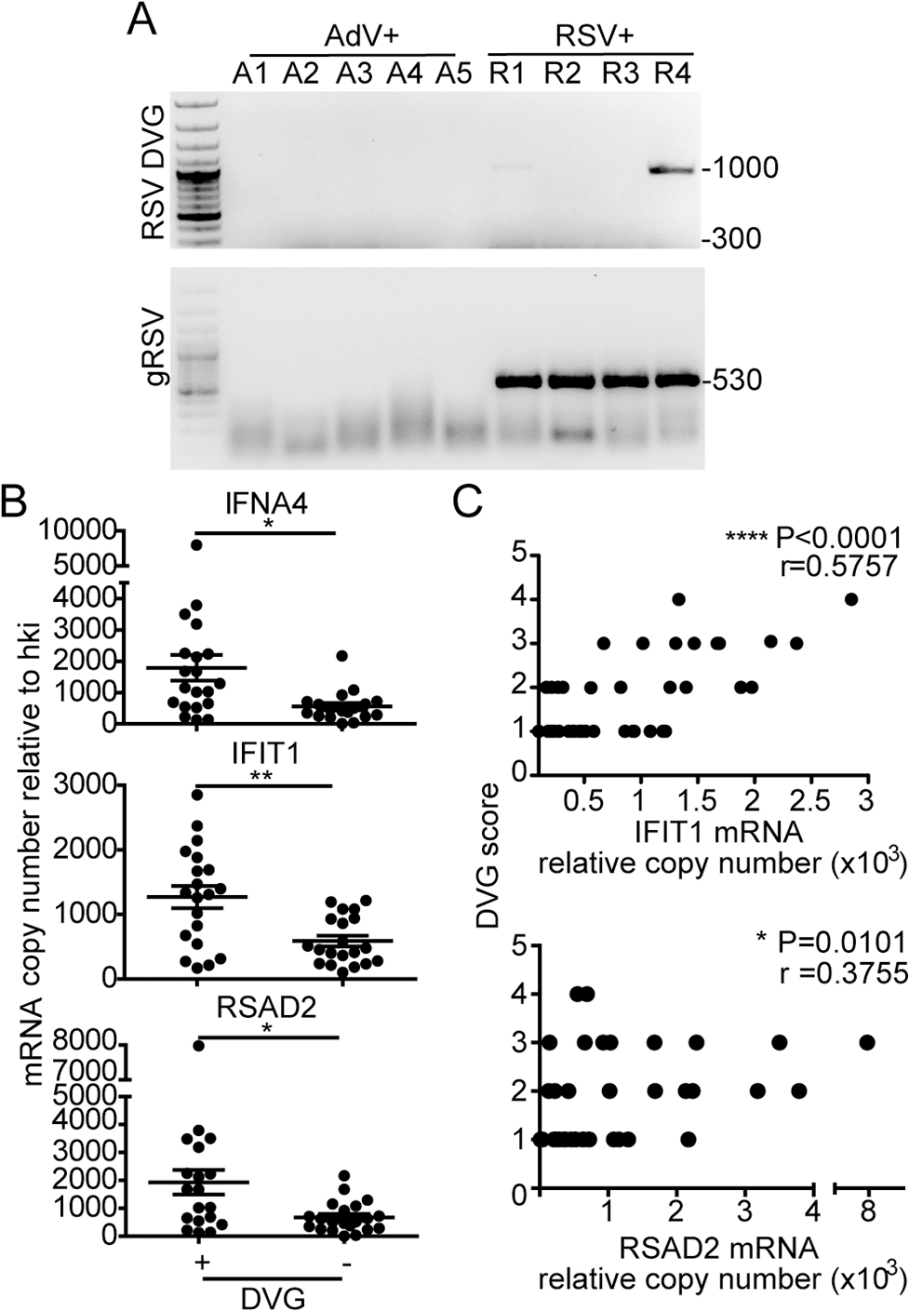
iDVGs associate with high expression of antiviral genes in respiratory secretions from patients infected with RSV. (A) Representative PCR results for gRSV and DVGs in human nasopharyngeal control samples infected with adenovirus (A1-A5) and samples infected with RSV (R1-R4). (B) Gene expression determined by RT-qPCR shown as copy number relative to house keeping genes (*p<0.05, **p<0.01, by two-tailed Mann Whitney test). (C) Samples were scored based on the intensity of the DVG amplicons (1–4, absent to highest intensity) and correlated with the level of expression of antiviral genes. (r = correlation coefficient, p<0.0001 for slope deviation from 0).

To determine if iDVGs are present in humans naturally infected with RSV and if they correlate with innate antiviral activity in respiratory samples of infected patients, we analyzed nasopharyngeal aspirates from pediatric patients with confirmed RSV infection. As controls for the specificity of genomic and DVG detection assays, we analyzed samples from patients infected with adenovirus (AdV). Only samples with comparable amounts of virus, as determined by their RT-qPCR cycle threshold (Ct) for genomic RSV (gRSV), and with sufficient amount of total cellular RNA for the full analysis were considered for the study (n = 41). While no gRSV or RSV DVGs were detected in samples from AdV-infected patients, DVGs were detected in 48.8% of the RSV positive samples (20/41) (Fig 5A, additional samples and quantification in S8 Fig). Remarkably, among the RSV positive samples, those with detectable levels of DVGs showed significantly higher expression of a number of antiviral genes including IFNA4, IFIT1, and RSAD2 (also known as viperin) (Fig 5B). Expression of these genes was positively correlated with the amount of DVGs detected as scored based on the intensity of the amplicon band in the PCR (Fig 5C). Notably, expression of IFNL1 and IFNB1 was not detectable in most of the patients (S9 Fig), likely because these primary genes are not longer expressed at high levels at the moment of sampling (when patients are very sick and go to the hospital) and only secondary ISGs can be measured. Of note, only patients admitted to the hospital and with equivalent levels of RSV genome were analyzed in this study. This study design reduced potential false negative results from samples that either contained very low levels of virus or viral RNA was degraded. This study did not consider the timing of infection during sampling, co-morbidities, previous or current treatment, or infection outcome. Studies with the appropriate patient populations need to be designed to evaluate these parameters. However, our data demonstrate that iDVGs are naturally generated during infections with RSV and indicate that iDVG accumulation correlates positively with the expression of genes with antiviral activity in patients.

### Host-intrinsic factors determine the quality of the response to iDVGs in the human lung

**Fig 6:**
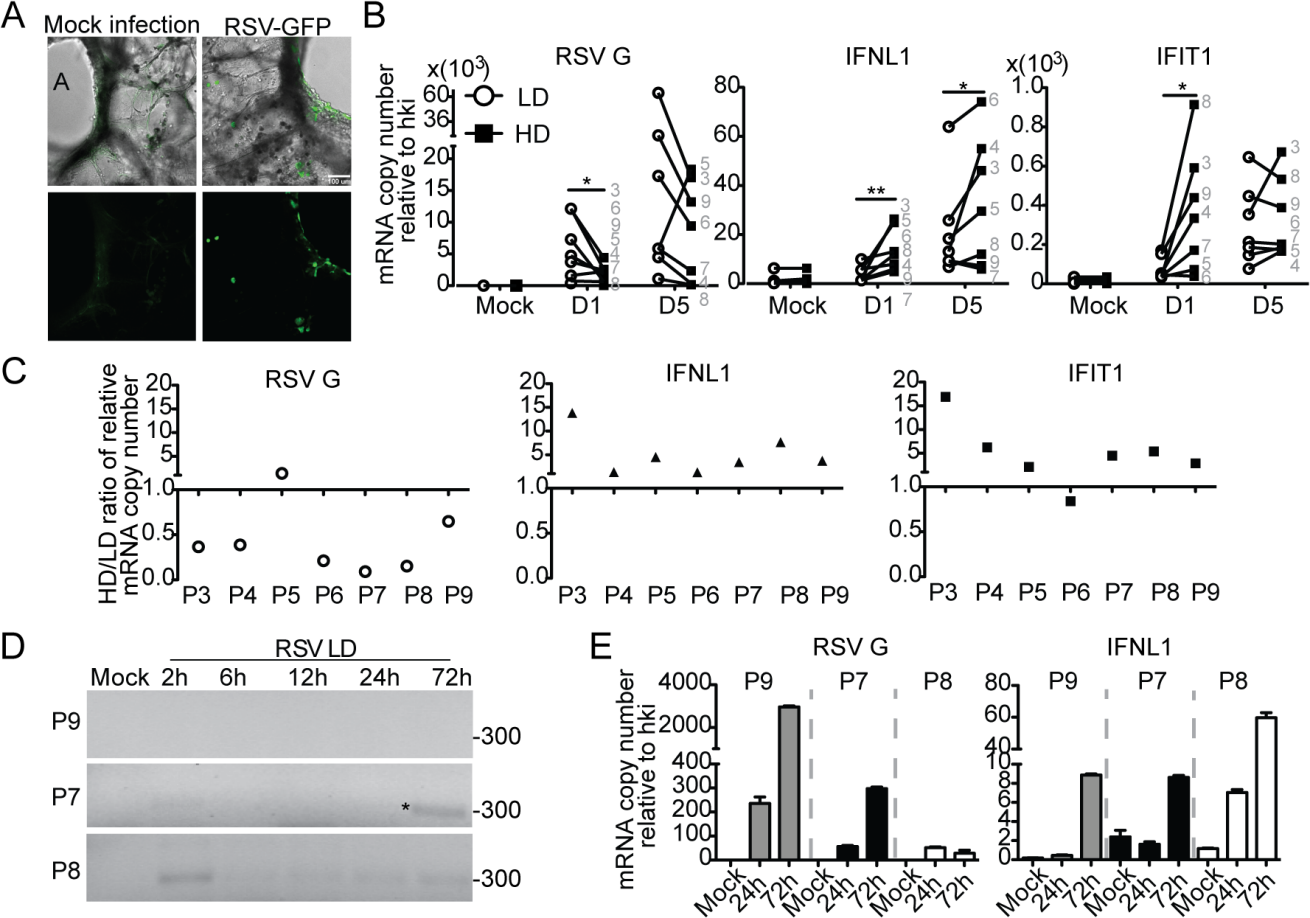
Host-intrinsic factors determine the response to iDVGs in the human lung. (A) Images of precision cut lung slices from human lungs (hPCLS) infected for 24 h with 10^6^ RSV-GFP TCID_50_/slice. Top: Overlay of bright field and fluorescence channels; Bottom: fluorescence channel alone. (B) Gene expression from hPCLS infected with 10^7^ TCID_50_/slice of RSV-LD or HD for up to 5 days (n = 7–8, grey numbers indicate individual lung donor). Results show paired data, numbers correspond to different donors. (*p<0.05, **p<0.01 by one-tailed Wilcoxon matched-pairs signed rank test). (C) Ratio of gene expression in RSV-HD and RSV-LD infected tissue from different donors (P3-P9). Ratio>1: HD induced a higher gene expression than LD. (D) PCR for DVGs in hPCLS infected with 10^6^ TCID_50_/slice of RSV-LD. (E) Gene expression from (D). Error bars indicate mean ± SEM of three slices from the same patient.

Failure to detect DVGs and heterogeneity of the response to DVGs in respiratory secretions from infected patients (Fig 5) could be a consequence of the timing of sampling (too early or late in the infectious cycle), virus intrinsic properties (such as virus strain and mutations), or to patient intrinsic properties (such as genetic determinants, co-morbidities, or co-infections). To establish whether host intrinsic factors contribute to the heterogeneity of the response to DVGs in humans, we infected precision cut lung slices prepared from lungs of human donors with no obvious disease. Infections of lungs from different donors were performed in identical conditions and using the same virus stocks to minimize extrinsic factors that may impact the outcome of infection. To validate this system, lung slices were first infected with RSV-GFP. As predicted from studies *in vivo*, GFP was expressed along the airway epithelium mimicking the natural virus distribution during infection (Fig 6A). Infection of human lung slices with RSV-HD consistently resulted in significantly reduced viral replication and enhanced expression of antiviral genes on day 1 post infection when compared to RSV-LD infections (Fig 6B and 6C and S10 Fig). This trend was maintained for some genes on day 5 post-infection while others were not significantly different at this time point, presumably due to the impact of restricted viral replication during RSV-HD infection. Interestingly, although some degree of heterogeneity in the responsiveness of different individuals to iDVGs was observed, lungs from six out of seven individuals tested responded to RSV-HD with higher expression of antiviral genes on day one post-infection (Fig 6C). Notably, in most donors the intensity of the response to iDVGs inversely correlated with viral replication during infection with RSV-HD compared to RSV-LD confirming the antiviral effect of iDVGs in humans.

To determine whether the rate of iDVG accumulation varied in different donors influencing the host antiviral response, we analyzed the kinetics of iDVG accumulation upon infection with RSV-LD. The buildup of iDVGs was strikingly different in lungs slices prepared from different donors directly impacting the rate of viral replication and the intensity of the antiviral response. Failure to generate iDVGs upon RSV-LD infection associated with 10 times higher expression of RSV G compared with fast iDVGs accumulation (Fig 6D and 6E). Notably, in one patient high levels of DVGs were detected at 2 h post infection corresponding with drastically reduced viral replication and stronger expression of antiviral genes (Fig 6D and 6E, P8). The source of these early DVGs is unclear, as it is unlikely that they would be the product of *de novo* generation during infection. It is possible that these DVGs were carried in the virus stock, or were amplified from persistent sources in the patient’s lung. Regardless, these data demonstrate that the rate of accumulation of DVGs in the lung is a critical determinant of virus growth and antiviral immunity. Additional samples showing a correlation between the generation of iDVGs and the IFN response in human lungs are shown in S11 Fig.

Overall, these data indicate that host factors influence the accumulation of RSV iDVGs in humans. Identification of these factors should allow for better prognosis of clinical outcome, as well as the development of strategies for modulating iDVG generation with therapeutic purposes.

## Discussion

DVGs have been identified in human infections with influenza virus, Dengue virus, HIV, and HCV [20–23], but until now, iDVGs have not been described in humans during natural *paramyxovirus* infections. Although a role for DVGs in interfering with the replication of the full-length viral genome has been known for decades, DVGs are frequently considered an epiphenomenon of *in vitro* viral replication and their biological role is neglected. This report is the first to demonstrate that iDVGs are naturally generated in humans during infection with a *paramyxovirus* and that they play a critical role in stimulating the antiviral response *in vitro* and *in vivo*. Together with our observations in mice [17], these studies suggest that iDVGs are essential immunostimulatory signals during *paramyxovirus* infections.

Studies in mice showed that lung pathology during RSV infection associated with poor and delayed expression of genes with antiviral function. This delayed innate response allowed for faster viral replication and the development of a highly neutrophilic inflammatory response. In contrast, in infections with a high content of iDVGs, viral replication was limited by a rapid and robust innate antiviral response that minimized weight loss and lung pathology. *In vitro*, RSV-HD and LD viral titers were no different in cells that failed to produce IFN, and *Ifnar-/-* MEFs showed enhanced replication of both LD and HD viruses compared to wild type cells, suggesting that IFNs induced by DVGs suppress viral growth. However, more studies are required to test whether DVGs may also directly impact the replication of standard virus *in vivo* through interference with the viral replication machinery. Although the impact of iDVGs on the development of RSV-specific CD4 and CD8 T cells was not directly evaluated in this study, our data show that an effective expression of antiviral genes early on can reduce damaging inflammatory cellular responses in the lung independent of T cell recruitment. It is noted that our RSV stocks induce a single early peak of weight loss in mice as has been reported by others [43, 44], but different from many reports of biphasic weight loss with a second peak at days 6–8 post infection [45–47]. We speculate that the original source of the virus or the method used for expansion over time may explain the observed differences in RSV pathogenicity in mice. Nevertheless, it is clear that iDVGs protect mice from RSV-induced pathology.

Despite of reports of a redundant role of type I and III IFNs [48], type III IFNs are the predominant IFNs induced by influenza in the lung of mice [49, 50] and IFNL1 is required to control infections by mucosal pathogens in humans [42, 51]. Our data support an essential role for type III IFNs in controlling respiratory viruses through a mechanism that involves the activation of IRF1. The precise molecular mechanisms mediating the recognition and potent response to iDVGs is intriguing given that the same MAVS-mediated sensing pathway is involved in the recognition of the full-length genome and this pathway is blocked by the RSV encoded NS1 and NS2 proteins [4, 30]. Notably, high levels of type I and III IFNs have been reported in a fraction of RSV-infected patients despite the encoded IFN antagonists [30–32, 52, 53], supporting the existence of mechanisms to bypass the virus antagonistic strategies. Important unresolved questions are how RSV iDVGs and standard viruses are differentially recognized by viral sensors and how sensing of iDVGs bypasses the antagonistic function of NS1 and NS2. One possibility is that iDVG RNA is more accessible to viral sensors that full-length genomes within the infected cell. Alternatively, it is possible to find cells primed by type I or III IFN and exposed to defective viral particles in the absence of standard virus genomes (and NS1/NS2 proteins). In this situation, iDVGs could trigger potent IFN independently of antagonism.

Using a uniquely suitable system for the *ex vivo* study of the impact iDVGs in the human lung, we demonstrate that RSV iDVGs promote the expression of antiviral genes in most donors and that iDVGs can control RSV replication in humans. The study of *ex vivo* lung infection revealed that host intrinsic factors play an important role in determining the kinetics of iDVG generation and their quantity, thereby affecting the innate control of viral replication. These factors may include age and sex of the patient, co-morbidities and ongoing medications. Remarkably, iDVGs were detected in >48% of patients admitted to the hospital with confirmed RSV infection. In this proof of principle study we analyzed samples with comparable viral loads that limited our ability to assess correlations between viral titers and DVG production. However, based on studies in mice and in human precision cut lung slices we predict that the rate of accumulation and amount of DVGs will determine the amount of virus in the patient and the clinical outcome. Early accumulation of DVGs would predict viral clearance and full disease recovery, while their delayed production or absence suggests poor engagement of host immune responses and the need for more aggressive treatments.

Why only a population of patients showed DVGs is unclear at the moment. It is possible that some patients sought medical help at a different stage of the infection or that iDVGs are detectable in respiratory secretions in a relatively narrow window of time after infection. Based on our studies *ex vivo*, it is also likely that iDVGs are only generated in a subpopulation of patients. In any case, detection of iDVGs on respiratory secretions sets the stage for a comprehensive evaluation of the impact of iDVGs in the clinical outcomes of infection with RSV, and suggests that modulating the generation and/or function of DVGs may provide a novel strategy for therapeutic intervention.

## Materials and Methods

### Ethics statement

Studies in mice were carried out in accordance with the recommendations in the Guide for the Care and Use of Laboratory Animals of the National Institutes of Health and approved by the Institutional Animal Care and Use Committee, University of Pennsylvania Animal Welfare Assurance Number A3079-01. De-identified human lung tissue from donors was obtained from the National Disease Research Interchange (NDRI), Philadelphia, PA. Analysis of human samples was approved by the University of Pennsylvania Internal Review Board.

### Cell lines and mice

HEp2 cells (HeLa-derived human epithelial cells, ATCC, #CCL23), A549 cells (human type II alveolar cells, ATCC, #CCL185), Vero cells (Cercopithecus aethiops kidney epithelial cells, ATCC, #CCL-81), D54MG cells (human glioma cells, kindly provided by Dr. Kathleen E. Sullivan), and wild type, *Mavs* ^-/-^ MEFs, *Ifnar-/-* MEFs were cultured in DMEM supplemented with 10% fetal bovine serum, 1 mM sodium pyruvate, 2 mL L-Glutamine, and 50 mg/ml gentamicin or penicillin and streptomycin. D54MG cells overexpressing IRF1(D54-IRF1) [54] were selected in the same culture medium with 20 μg/ml puromycin. All cell lines were treated with mycoplasma removal agent (MP Biomedicals) before use. BALB/C mice were obtained from Taconic. All experiments used female mice of 6–8 weeks of age.

### Human lung tissue and nasopharyngeal aspirates

Human precision cut lung slices were prepared as previously described [55]. In brief, whole human lungs from donors were inflated using 2% (wt/vol) low melting point agarose. The lungs were then cored to include a small airway and sliced at a thickness of 300 μm (VF300 Vibratome). The slices were incubated at 37°C in a humidified air-CO_2_ (95–5%) incubator and stock media (Ham’s F-12 with 100 U/ml penicillin, 0.1 mg/ml streptomycin, and 2.5 mg primocin) was replaced every 2–3 h for 1–2 days to remove agarose.

Nasopharyngeal aspirates from pediatric patients were obtained from banked samples at the Clinical Virology Laboratory of Children’s Hospital of Philadelphia. Samples were obtained as part of standard testing for patients admitted to the hospital between the years 2012–2014. Samples were de-identified and RNA was extracted and processed in our laboratory as described below.

### Viruses

RSV strain A2 (ATCC, #VR-1540) and RSV-GFP (kindly provided by Dr. Mark Peeples, Nationwide Children’s Hospital, Columbus, OH) were propagated in HEp2 cells in our laboratory. HEp2 cells were inoculated with RSV-GFP at a multiplicity of infection (moi) of 1.5 pfu/cell and incubated at 37°C for 2 h. The inoculum was then removed and fresh media (DMEM supplemented with 10% fetal bovine serum, 1 mM sodium pyruvate, 2 mL L-Glutamine, and 50 mg/ml gentamicin or penicillin and streptomycin) was added. Viruses were harvested 1–2 days post infection, when nearly all the cells expressed GFP. For propagation of RSV A2, cells were inoculated with a moi of 0.01 medium tissue culture infectious dose (TCID_50_)/cell and viruses were harvested 5 days post infection. Stocks of RSV depleted of DVGs (RSV-LD) were generated after two rounds of RSV A2 expansion at a moi of 0.01 TCID_50_/cell for five days each. Stocks of RSV with a high content of DVGs (RSV-HD) were generated after two rounds of RSV A2 expansion at a moi of 4 TCID_50_/cell followed by one to two rounds of expansion at a moi of 10 TCID_50_/cell for two days each. Supernatants from infections were centrifuged at 15,000 rpm for 2.5 h to concentrate the virus prior to aliquoting and freezing in dry ice/ethanol for storage.

### Virus titration and infections

For titration, HEp2 cells were infected with triplicate serial 1:10 dilutions of virus stock or 1:2 dilutions of lung homogenates in the presence of 2% FBS. After 4–5 days of incubation in 7% CO_2_ at 37°C end point dilution titer (TCID_50_) was determined by crystal violet staining of the monolayers_._ For infections *in vitro*, cells were incubated with virus at a multiplicity of infection of 1.5 TCID_50_/cell. For infections *ex vivo*, human lung slices were infected with 10^6^ or 10^7^ TCID_50_/slice in Ham’s F-12 medium supplemented with 100 U/ml penicillin (Cellgro), 0.1 mg/ml streptomycin (Cellgro), and 2.5 mg/ml primocin (Invitrogen). Medium was replaced 24 h post-infection. For infections *in vivo*, mice were anesthetized with Ketamine HCI (Ketaset) and Xylazine (VEDCO) and inoculated intranasally with 35 μl of PBS containing 5 x 10^6^ medium tissue culture infectious dose of RSV. Lungs were extracted at different times post-infection, homogenized in 0.1% w/v Gelatin-PBS and snap frozen in dry-ice/ethanol for preservation.

### RNA interference

siRNAs for human IRF1 (3659, ON-TARGETplus smart pool including 4 target sequences: GGGCUCAUCUGGAUUAAUA, UGAACUCCCUGCCAGAUAU, GCUCAGCUGUGCGAGUGUA, GAAGGGAAAUUACCUGAGG), siRNA for human IRF3 (3661, ON-TARGETplus smart pool including 4 target sequences: CGAGGCCACUGGUGCAUAU, CCAGACACCUCUCCGGACA, GGAGUGAUGAGCUACGUGA, AGACAUUCUGGAUGAGUUA) and ON-TARGETplus non-targeting control pool were purchased from GE health, Dharmacon. 3 x 10^4^ A549 cells were transfected with 25 μM of different siRNAs with Lipofectamine RNAiMAX complexes (Invitrogen) according to the manufacture’s instruction (reverse transfection). After 16 h of incubation, media was replaced by complete cell culture media without antibiotics. After 40 h of transfection, cells were infected with RSV-HD at a moi of 1.5 TCID_50_/cell for 10 h. Cells were harvest with either TRIzol for RNA or NP-40 lysis buffer for protein analysis. As control, cells were treated only with Lipofectamine RNAiMAX transfection reagent.

### RNA extraction and PCR for DVGs and viral genome detection

Total RNA was extracted from cell lines, human lung slices, or mice lungs with TRIzol (Invitrogen) according to the manufacturer’s specifications. For pediatric nasopharyngeal samples, total RNA was extracted with TRIzol LS (Invitrogen). For detection of DVGs, isolated total RNA was reverse transcribed with the primer 5’CTTAGGTAAGGATATGTAGATTCTACC3’ using the SuperScript III reverse transcriptase (Invitrogen) without RNase H activity to avoid self-priming. Recombinant RNase H (Invitrogen) was later added to the reverse transcribed samples and incubated for 20 min at 37°C. DVGs were partially amplified using the primers: *for*-5’CCTCCAAGATTAAAATGATAACTTTAGG3’ and *rev*-5’CTTAGGTAAGGATATGTAGATTCTACC3’. For detection of standard viral genome, RNA was reverse transcribed with the same kit using the primer 5’GATAAATATAGGCATGGGGAAAGTG3’. Amplification of the intergenomic segment between the *ns1* and *ns2* genes was performed using the primers: *for*-5’CACTGCTCTCAATTAAACGGTCTA3’ and *rev*-5’GATAAATATAGGCATGGGGAAAGTG3’. The temperature cycle parameters used for the PCR in a BioRad C1000 Thermal Cycler were: 95°C for 10 min and 33–35 cycles of 95°C for 30 sec, 55°C for 30 sec and 72°C for 90 sec followed by a hold at 72°C for 5 min. Ramp rate of all steps was at 3 degree/sec.

### RT-qPCR

Total RNA was reversed transcribed using the high capacity RNA to cDNA kit from Applied Biosystems. For pediatric nasal secretions, 500 ng of RNA were reversed transcribed, for all other experiments 1–2 μg of RNA were reversed transcribed. cDNA was diluted to a concentration of 10 μg/μl and amplified with specific primers in the presence of SYBR green (Applied Biosystems). qPCR reactions were performed in triplicate using specific primers and the Power SYBR Green PCR Master Mixture (Applied Biosystems) in a Viia7 Applied Biosystems Lightcycler. Normalization was conducted based on levels of ACTB and GAPDH for human samples and *Rsp11* and α*-tubulin* for mice samples. Sequences of primers used in these studies can be found in the supplementary materials S1 Table.

### RSV defective particles (DPs) purification

Concentrated RSV-HD was loaded in a 20%-60% sucrose in PBS/2 mM EDTA gradient and centrifuged at 23,000 rpm for 2 h at 4°C. Fractions containing low-density viral particles were collected, pelleted, suspended, and re-purified using the same procedure followed by concentration by centrifugation at 4°C for 2 h at 21,000 rpm. Pellets were suspended in 2% Gelatin in PBS, snap frozen, and stored at -80°C. The content of DPs particles was determined by calculating the ratio of infectious particles over total proteins as determined by Bradford assay (Thermo Scientific).

### Immunofluorescence assay and imaging

Cells were seeded on coverslips and infected with RSV-LD and RSV-HD as described above. At designated times post infection, cells were fixed with 4% paraformaldehyde and permeabilized with 0.2% Triton-X 100, followed by incubation with IRF-1 rabbit mAb (D5E4, Cell Signaling) and serum from mice immunized with RSV F + G (kindly provided by David Weiner, UPenn). Slides were then washed and incubated with secondary antibodies (goat anti-rabbit Alexa Fluor 488 and goat anti-mouse Alexa Fluor 594, Life Technologies), Hoechst stained, and mounted onto slides with Fluoromount-G (eBiosciences). Slides were imaged on a Nikon E600 Widefield microscope at 40X. Analysis was performed by capturing 5 different fields per slide to total approximately 150 cells per slide. Exposure time, gain, and offset were held constant for all images. Quantification of nuclear-localized IRF1 was performed on the Volocity software by setting a threshold of detection for nuclear accumulation based on mock-infected samples. Nuclear IRF1-positive cells among the whole population were identified using the “intersect module” with IRF1 and Hoechst. Human lung slices were imaged under 37°C in stock medium at 10X magnification on a Leica DMI4000 inverted microscope with a Yokagawa CSU-X1 spinning disk confocal attachment controlled by Metamorph software.

### Cell fractionation and western blot

A549 cells were seeded in 6-well plates one night before infection. Cells were mock infected or infected with RSV-LD, RSV-HD at moi of 1.5 TCID_50_/cell. At 2 h, 6 h, 12 h, and 24 h post infection, cells were fractionated using the nuclear/cytosol fractionation kit (BioVision Technologies) according to the manufacturer’s instructions. Briefly, cells were collected in 1 ml cold PBS per well and centrifuged at 4°C for 5 min at 600 x g in a microcentrifuge. Cell pellets were resuspended in CEB (cytosolic extraction buffer)-A, and incubated for 10 min on ice prior to addition of CEB-B. The lysates were centrifuged at 4°C for 5 min at 12,000 rpm in a microcentrifuge and the supernatants were kept as a cytoplasmic fraction. The nuclear pellet was resuspended in NEB (nuclear extraction buffer) and vortexed for 30 s. This step was repeated 5 times 10 min each. The nuclear pellet was centrifuged at 4°C for 10 min with 12,000 rpm and the supernatants were kept as a nuclear fraction. The cytoplasmic and nuclear fractions were resolved by SDS-PAGE followed by western blot. For western blot, both the whole cells lysate and the nuclear/cytosolic fractions were used. The whole cellular extracts were prepared by lysing 3 x 10^5^ of cells in a NP-40-based lysis buffer containing phosphatase inhibitors, proteinase inhibitors (Roche and Thermo Scientific). The concentration of protein was measured by Bradford assay or BCA assay (Themo Scientific). Samples (10–20 μg) were boiled for 5 min and resolved on 10% Bis-Tris precast gels (Bio-rad). Resolved proteins were transferred to a polyvinylidene fluoride (PVDF) membrane (Millipore). The membrane was blocked with 5% non-fat milk and immunoblotted with the indicated antibodies. Anti-rabbit IRF1, anti-rabbit IRF3, and anti-rabbit IgG (HRP-conjugated) were purchased from Cell Signaling. Anti-mouse GAPDH was purchased from Sigma. Anti-mouse IgG and anti-mouse IgG_1_ (HRP-conjugated) were purchased from Jackson Immunologicals. Anti-rabbit Histone 3 was purchased from Abcam. Lumi-Light western blotting substrate was used for HRP detection (Roche).

### Histology

After lavage, the left lobe of the lung was inflated and fixed with 0.5 ml of 10% neutral-buffered formalin solution. Deparaffinized sections from fixed lungs were stained with hematoxylin and eosin (H&E). Lung infiltration was scored blindly according taking in consideration the amount of inflammation and the frequency of inflamed foci in the lung, and following scale: peribronchiolotis (0 none to 4 severe), alveolitis (0 none to 4 severe), vasculitis (0 none to 4 severe), epithelial cell hypertrophy (0 none to 4 severe).

### Flow cytometry

Lungs were flushed with media containing 2 mM L-Glutamine, 10% FBS, 0.2% β-mercaptoethanol, 2% Pen/Strep, 1% Liberase Blendzymes (Roche) and incubated at 37°C for 40 min. Cells were pelleted and red blood cells were lysed. Single-cell suspensions were incubated with anti-mouse CD16/32 (BD Bioscience) for 10 min at 4°C. The following antibodies from BD Bioscience, eBioscience, or Serotec were used for staining in two panels and incubated on ice for 20 min. Panel 1: CD11b (M1/70), Ly6G (1A8), Ly6C (HK1.4), F4/80 (BM8), and CD45 (104), Aqua. Panel 2: CD4, CD8, CD3, NKp46, CD19, Life/Dead Aqua. Flow cytometry was performed in a BD FACSCanto II Flow cytometer.

### Cytospins

Bronchioalveolar lavage (BAL) was performed by instilling and collecting 1 ml of sterile saline into the lungs of euthanized mice. BAL cells were pelleted and counted and 5x10^4^ cells in 200 μl PBS were centrifuged in a Cytospin. Preparations were air dried overnight and then and stained using Kwik-Diff (Thermo Scientific). Pictures were visualized in a Nikon Eclipse E600 microscope.

### ELISA

IFNβ in the cell-free fraction of the bronchoalveolar lavage was determined by ELISA following the manufacturer’s instructions (PBL Assay Science). IFNB1 and IFNL1/3 in the supernatants from RSV-LD or RSV-HD infected A549 cells were measured by human IFNB1 and human IFNL1/3 ELISA kit following the manufacturer’s instructions (PBL assay Science).

### Statistical analysis

Statistical analyses were performed as indicated in each Fig. All data were included in the analysis. GraphPad Prism version 5.00 (San Diego California USA, www.graphpad.com) was used for analysis.

### Genes NCBI ID numbers

For mice, *Tuba1b*: 22143; *Rps11*:27207; *Ifnb*: 15977; *Ifnl2*: 330496; *Il1b*: 16176; *Il1a*: 16175; *Tnf*: 2926; *Il-6*, 16193. For human, ACTB: 60; GAPDH: 2597; IFNB1: 3456; IFNL1: 282618; RSAD2: 91543; IFIT1: 3434; IRF1: 3659; IRF3: 3661. RSV G: 3089371.

## Supporting Information

S1 Fig RSV copy-back DVG PCR strategy and validation. (A) Diagram of the genomic composition of the full-length RSV genome (gRSV) and of a representative copy-back DVG of unknown length. Arrows indicate the location of primers used for RT and amplification (PCR). Full-length size of the genome and expected amplicon size of 530 nt of the gRSV to be detected through our PCR assay is indicated. This strategy allows detection of copy-back DVGs of various sizes as indicated by the interrogation sign. (B) Schematics of the 670 nt-long DVG from RSV. Expected amplicon size of 271 nt is indicated. (C-D) Validation of the DVG PCR assay. (C) To examine the primer sets, PCR for gRSV and DVG were performed using RNA extracted from HEp2 cells infected with RSV-LD. Amplicon of RSV genome and DVG were observed at 530 nt and at 271 nt, separately, as expected. (D) To test for the specificity of the DVG primers, PCR for gRSV and DVG were performed from a plasmid encoding the full-length RSV genome (pRSV). The expected 530 nt-long amplicon was produced using the gRSV primer set and no products were amplified using the DVG primer set, as expected.
(TIF)

S2 Fig Representative sequences of RSV DVGs. (A) Consensus sequence of a low molecular weight DVG that arises during infection of HEp2 cells or human explanted lung slices with a multiplicity of infection of 1.5 medium tissue culture infectious dose /cell or 10^6^ pfu/slice of RSV-LD, respectively. Sequence is shown as cDNA. (B-E) Sequences of the amplicon of high molecular weight DVGs found during the infection with RSV-HD. All these sequences refer to Fig 1 and Fig 2 of the manuscript.
(TIF)

S3 Fig Histology scores of peribronchial and perivascular infiltration. Balb/c mice were infected intranasally with 5 x 10^6^ TCID_50_/mouse of RSV-LD or HD, or mock infected. Both peribronchial and perivascular infiltration were scored based on H&E staining (n = 6 mice per group).
(TIF)

S4 Fig Flow gating for neutrophil, macrophages, T cell, B cells, and NK cells. (A) Gating of cell populations shown in Fig 1H of the manuscript. (A) For the analysis of myeloid cells, the population was pre-gated for singlets, live, and CD45^+^CD11b^+^ cells. Neutrophil (PMN) were identified as Ly6G^hi^Ly6C^lo^ cells, alveolar macrophages (Alv. Macs) as CD11b^intermediate (Int)^F4/80^+^ and monocytes (mono) as Ly6C^hi^. (B) For the analysis of lymphocytes, the population was pre-gated for singlets, live, and CD3^+^ cells. T cells were identified based of the expression of CD4 and CD8. B cells were identified as CD3^-^CD19^+^. NK cells were identified as CD3^-^NKp46^+^.
(TIF)

S5 Fig Infectivity of RSV-LD and RSV-HD stocks. Vero cells were infected with RSV-LD and RSV-HD at a moi of 1.5 or 0.5 TCID_50_ /cell. (A) mRNA expression of RSV G, (B) TCID_50_ in infected cells, (C) DVGs detected by PCR at the indicated time points.
(TIF)

S6 Fig Antiviral genes expression in A549 cells infected with RSV-HD. A549 cells were seeded in 6-well plate containing slides and then infected with RSV-HD at moi of 1.5 TCID_50_/cell. At various time points post infection, slides were fixed for IFA. A sample of cells from each well was collected to test for gene expression. Gene expression is shown as copy number relative to the hki. All error bars indicate mean ± SEM of three independent experiments (*p<0.05, **p<0.01, ***p<0.001, ****p<0.0001 by two-way ANOVA with Bonferroni post hoc test).
(TIF)

S7 Fig IRF3 is required for IRF1 induction. A549 cells were mock transfected (WT) or transfected with control siRNA (si-C), IRF1 siRNA (si-1), or IRF3 siRNA (si-3). After 40 h, the cells were mock infected or infected with RSV-HD at moi of 1.5 TCID_50_/cell. (A) WB for IRF3 and IRF1 was performed to confirm specific knockdown of IRF1 protein. (B) Expression of IRF1 and IRF3 at 10 h post virus infection. Gene expression is shown as copy number relative to GAPDH. All error bars indicate mean ± SEM of at least three independent experiments (*p<0.05, **p<0.01, ***p<0.001, ****p<0.0001 by two-way ANOVA with Bonferroni post hoc test).
(TIF)

S8 Fig Additional representative gel pictures of DVG positive pediatric secretions. (A) RNA was extracted from pediatric secretions using TRIzol LS according to the manufacturer’s instructions. 0.5 μg of total RNA was used for RT-DI-PCR as illustrated in S1 Fig and materials and methods. Whole PCR products were applied to agarose gel electrophoresis. (B) Quantification of intensity of all DVG bands amplified from pediatric secretions shown in the manuscript. DVG bands resulting from PCR amplification were quantified using Image J software. For positive samples DVG bands were normalized to the background (negative samples) on each gel and a ratio of intensity/background was calculated. No bands: score 1; ratio < 50: score 2; ratio 50–300: score 3; ratio > 300: score 4.
(TIF)

S9 Fig Expression of IFNL1 and IFNB1 in respiratory samples. Gene expression determined by RT-qPCR shown as copy number relative to house keeping gene expression index determined from ACTB and GAPDH. Samples were scored based on the intensity of the DVG amplicons (1–4, absent to highest intensity) and correlated with the level of expression of IFNL1 and IFNB1. (r = correlation coefficient, p<0.0001 for slope deviation from 0).
(TIF)

S10 Fig Additional antiviral genes in hPCLS infected with RSV-HD or LD. hPCLS were infected with 10^7^ TCID_50_/slice RSV-LD or RSV-HD and kept in culture for 5 days. Slices from 7 different donors were tested for expression of RSV G or antiviral genes mRNA by qRT-PCR. Lung slices from patient 1 and 2 were used for setting up the system. Gene expression is shown as copy number relative to a house keeping gene expression index determined from ACTB and GAPDH. Results showed paired data, numbers represent the corresponding patient lung slice. (*p<0.05, **p<0.01 by one-tailed Wilcoxon matched-pairs signed rank test).
(TIF)

S11 Fig Additional data on DVGs accumulation in hPCLS infected with RSV-LD. hPCLS were infected with 10^6^ TCID_50_/slice RSV-LD and kept in culture for up to 3 days. Slices from 2 different donors were tested for DVGs by DI-RT-PCR (A) and expression of RSV G or antiviral genes mRNA by qRT-PCR (B). Error bars indicate mean ± SEM of three slices from the same patient.
(TIF)

S1 Table RT-qPCR primer list. Names in uppercase correspond to genes in humans and names in lowercase indicate genes from mice.
(DOC)
