# Supplementary figures and images for: Immunostimulatory Defective Viral Genomes from Respiratory Syncytial Virus Promote a Strong Innate Antiviral Response during Infection in Mice and Humans
Source: PLoS Pathog. 2015 Sep 3;11(9):e1005122. doi: 10.1371/journal.ppat.1005122 (PMC4559413; doi:10.1371/journal.ppat.1005122)

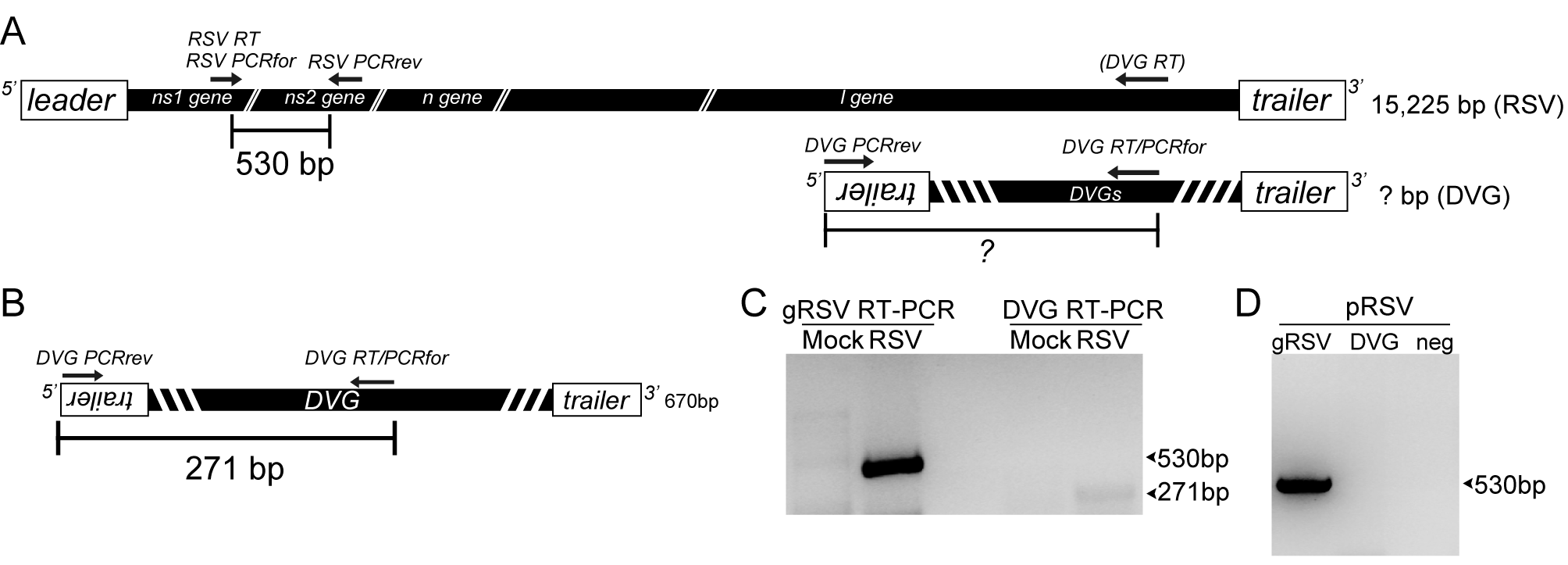

Supplement: S1 Fig — (A) Diagram of the genomic composition of the full-length RSV genome (gRSV) and of a representative copy-back DVG of unknown length. Arrows indicate the location of primers used for RT and amplification (PCR). Full-length size of the genome and expected amplicon size of 530 nt of the gRSV to be detected through our PCR assay is indicated. This strategy allows detection of copy-back DVGs of various sizes as indicated by the interrogation sign. (B) Schematics of the 670 nt-long DVG from RSV. Expected amplicon size of 271 nt is indicated. (C-D) Validation of the DVG PCR assay. (C) To examine the primer sets, PCR for gRSV and DVG were performed using RNA extracted from HEp2 cells infected with RSV-LD. Amplicon of RSV genome and DVG were observed at 530 nt and at 271 nt, separately, as expected. (D) To test for the specificity of the DVG primers, PCR for gRSV and DVG were performed from a plasmid encoding the full-length RSV genome (pRSV). The expected 530 nt-long amplicon was produced using the gRSV primer set and no products were amplified using the DVG primer set, as expected. (TIF) [file ppat.1005122.s001.tif]

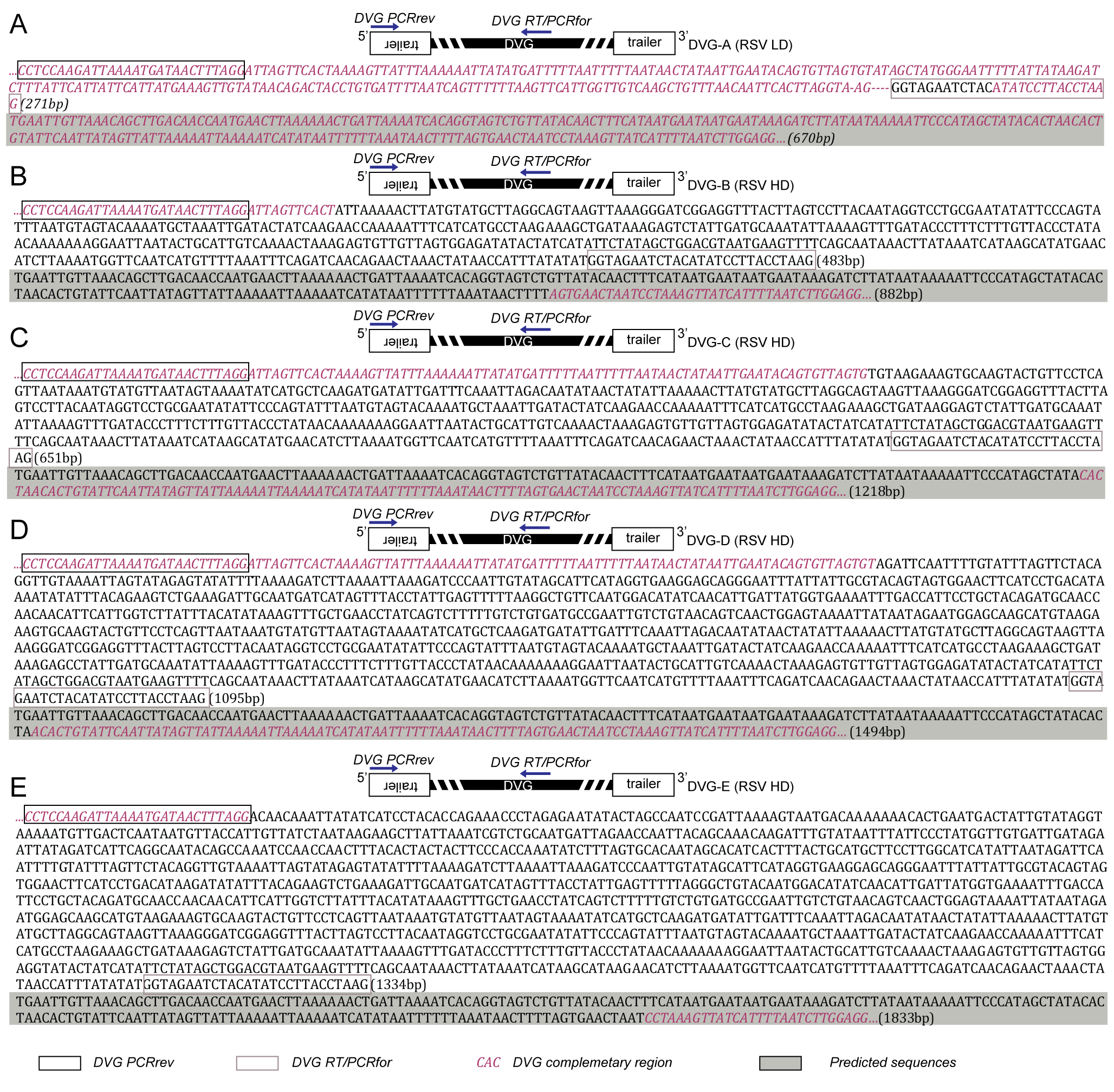

Supplement: S2 Fig — (A) Consensus sequence of a low molecular weight DVG that arises during infection of HEp2 cells or human explanted lung slices with a multiplicity of infection of 1.5 medium tissue culture infectious dose /cell or 106 pfu/slice of RSV-LD, respectively. Sequence is shown as cDNA. (B-E) Sequences of the amplicon of high molecular weight DVGs found during the infection with RSV-HD. All these sequences refer to Fig 1 and Fig 2 of the manuscript. (TIF) [file ppat.1005122.s002.tif]

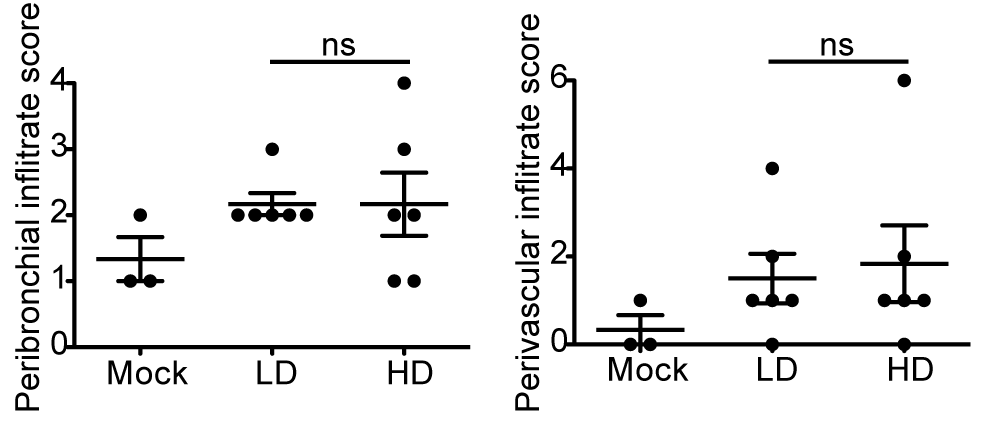

Supplement: S3 Fig — Balb/c mice were infected intranasally with 5 x 10^6^ TCID50/mouse of RSV-LD or HD, or mock infected. Both peribronchial and perivascular infiltration were scored based on H&E staining (n = 6 mice per group). (TIF) [file ppat.1005122.s003.tif]

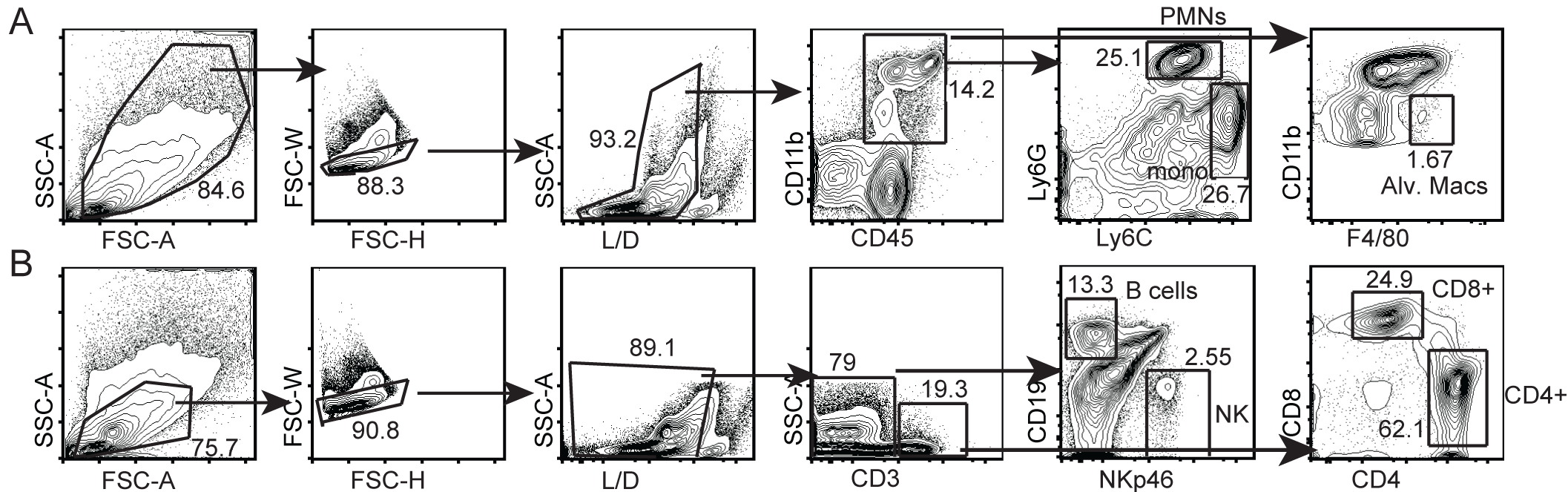

Supplement: S4 Fig — (A) Gating of cell populations shown in Fig 1H of the manuscript. (A) For the analysis of myeloid cells, the population was pre-gated for singlets, live, and CD45+CD11b+ cells. Neutrophil (PMN) were identified as Ly6GhiLy6Clo cells, alveolar macrophages (Alv. Macs) as CD11bintermediate (Int)F4/80+ and monocytes (mono) as Ly6Chi. (B) For the analysis of lymphocytes, the population was pre-gated for singlets, live, and CD3+ cells. T cells were identified based of the expression of CD4 and CD8. B cells were identified as CD3-CD19+. NK cells were identified as CD3-NKp46+. (TIF) [file ppat.1005122.s004.tif]

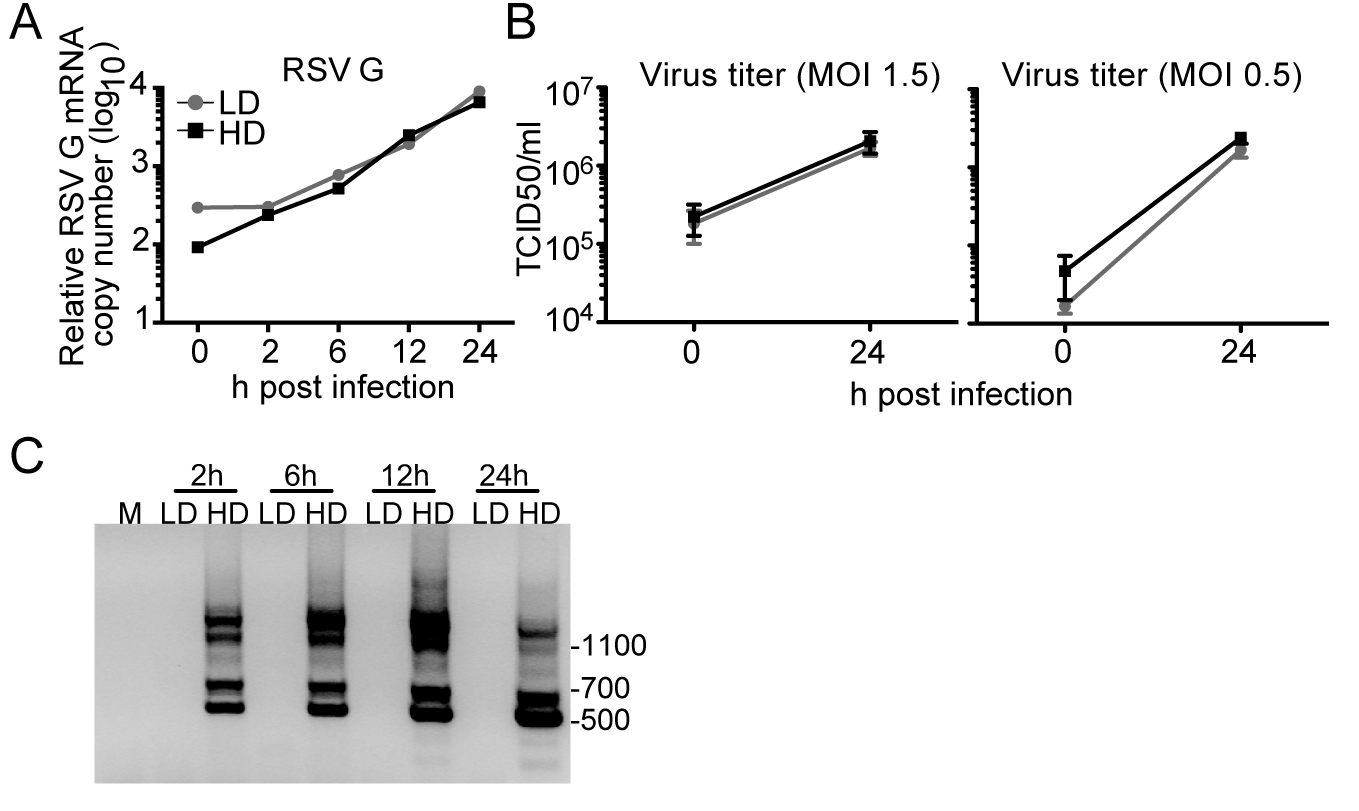

Supplement: S5 Fig — Vero cells were infected with RSV-LD and RSV-HD at a moi of 1.5 or 0.5 TCID50 /cell. (A) mRNA expression of RSV G, (B) TCID50 in infected cells, (C) DVGs detected by PCR at the indicated time points. (TIF) [file ppat.1005122.s005.tif]

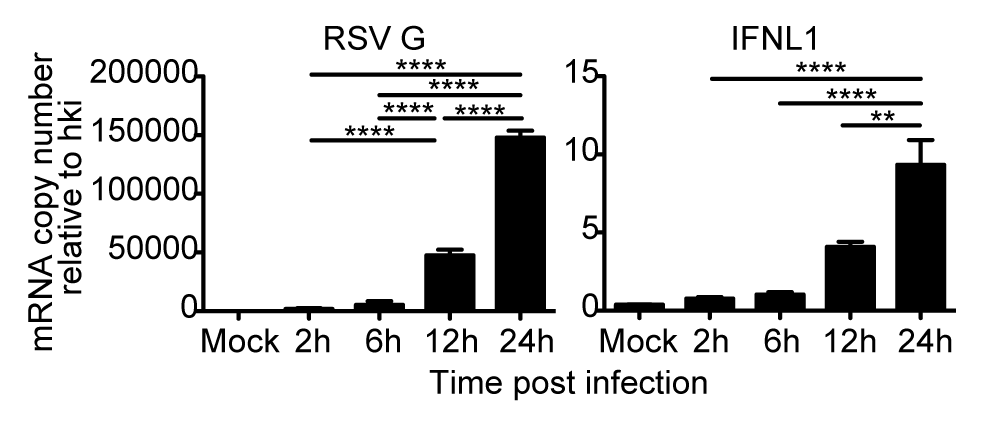

Supplement: S6 Fig — A549 cells were seeded in 6-well plate containing slides and then infected with RSV-HD at moi of 1.5 TCID50/cell. At various time points post infection, slides were fixed for IFA. A sample of cells from each well was collected to test for gene expression. Gene expression is shown as copy number relative to the hki. All error bars indicate mean ± SEM of three independent experiments (*p<0.05, **p<0.01, ***p<0.001, ****p<0.0001 by two-way ANOVA with Bonferroni post hoc test). (TIF) [file ppat.1005122.s006.tif]

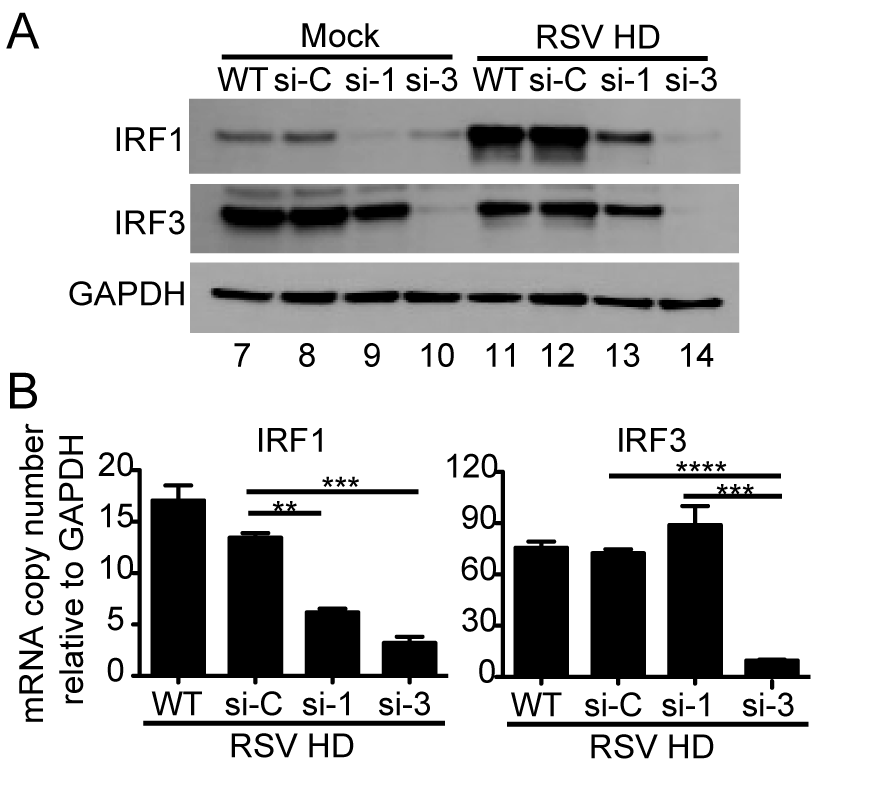

Supplement: S7 Fig — A549 cells were mock transfected (WT) or transfected with control siRNA (si-C), IRF1 siRNA (si-1), or IRF3 siRNA (si-3). After 40 h, the cells were mock infected or infected with RSV-HD at moi of 1.5 TCID50/cell. (A) WB for IRF3 and IRF1 was performed to confirm specific knockdown of IRF1 protein. (B) Expression of IRF1 and IRF3 at 10 h post virus infection. Gene expression is shown as copy number relative to GAPDH. All error bars indicate mean ± SEM of at least three independent experiments (*p<0.05, **p<0.01, ***p<0.001, ****p<0.0001 by two-way ANOVA with Bonferroni post hoc test). (TIF) [file ppat.1005122.s007.tif]

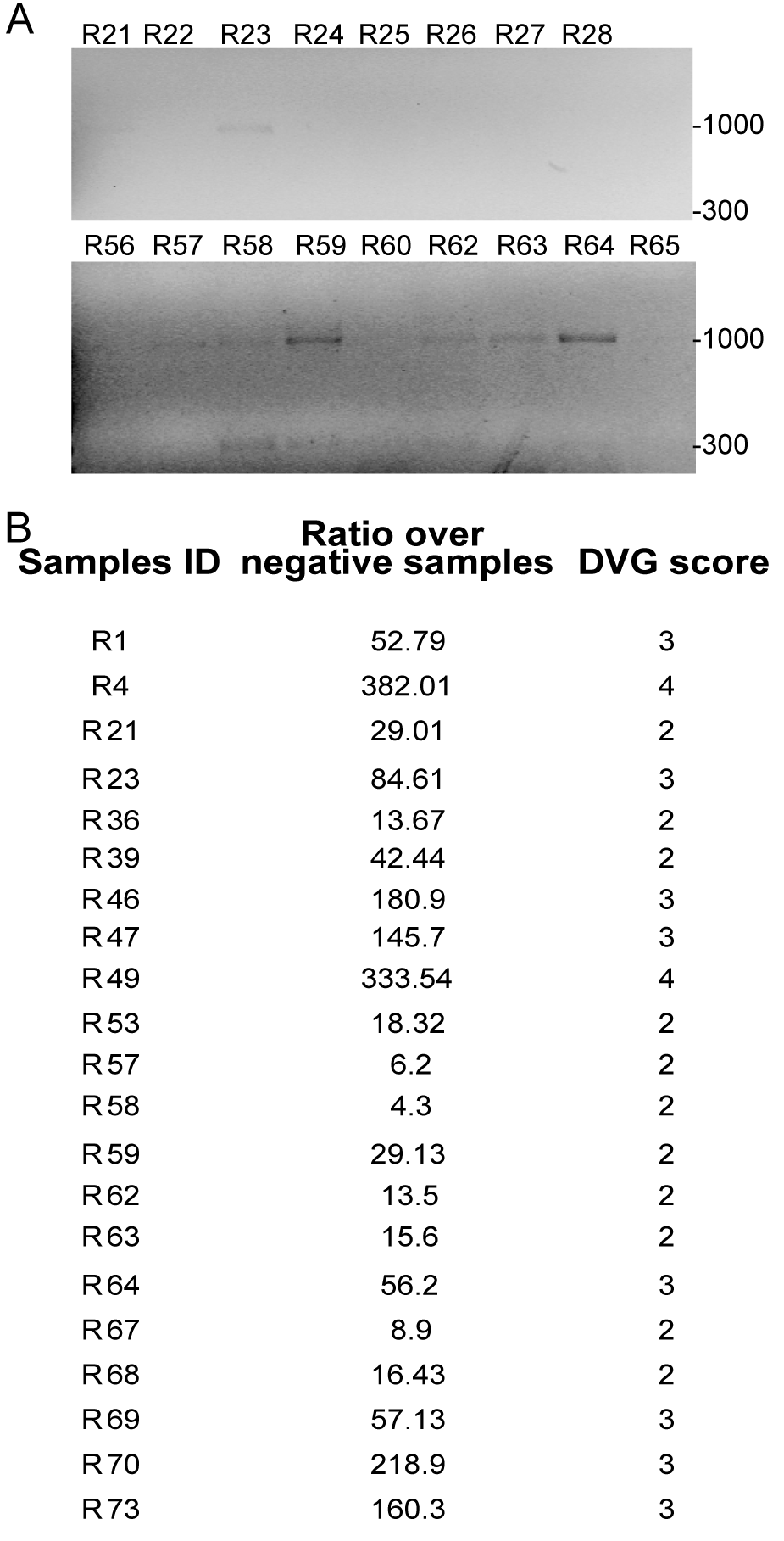

Supplement: S8 Fig — (A) RNA was extracted from pediatric secretions using TRIzol LS according to the manufacturer’s instructions. 0.5 μg of total RNA was used for RT-DI-PCR as illustrated in S1 Fig and materials and methods. Whole PCR products were applied to agarose gel electrophoresis. (B) Quantification of intensity of all DVG bands amplified from pediatric secretions shown in the manuscript. DVG bands resulting from PCR amplification were quantified using Image J software. For positive samples DVG bands were normalized to the background (negative samples) on each gel and a ratio of intensity/background was calculated. No bands: score 1; ratio < 50: score 2; ratio 50–300: score 3; ratio > 300: score 4. (TIF) [file ppat.1005122.s008.tif]

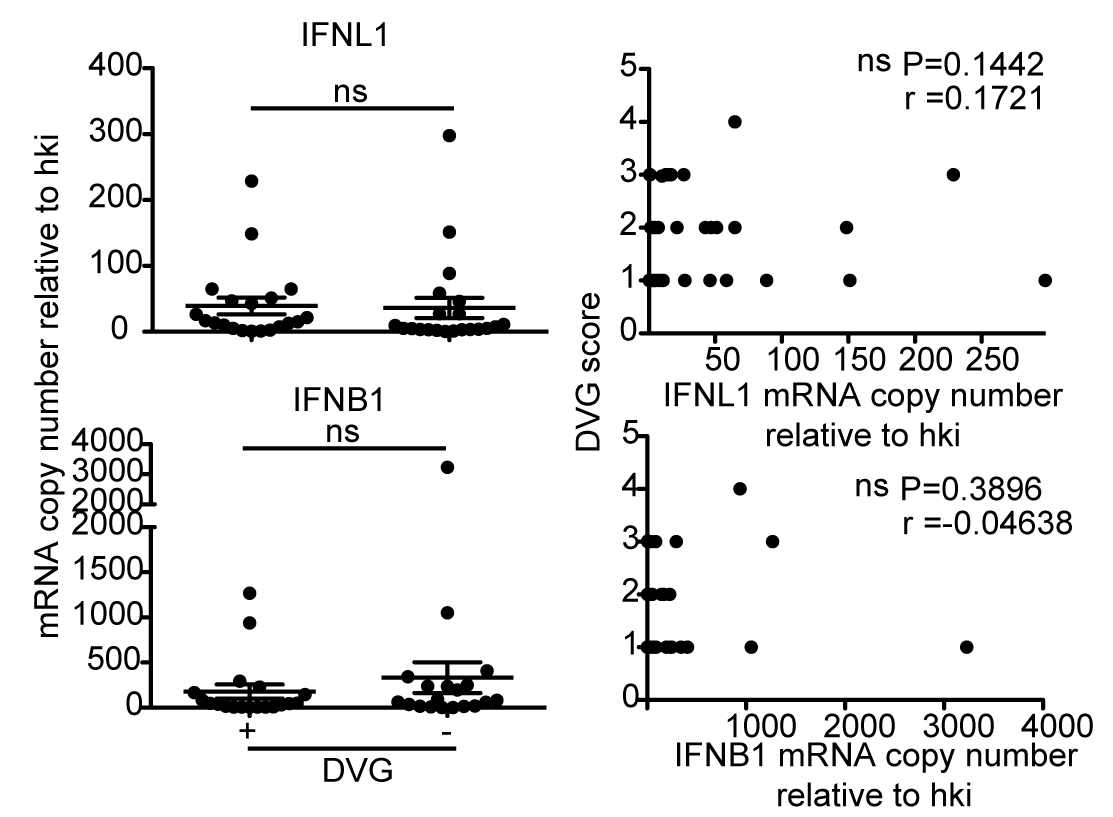

Supplement: S9 Fig — Gene expression determined by RT-qPCR shown as copy number relative to house keeping gene expression index determined from ACTB and GAPDH. Samples were scored based on the intensity of the DVG amplicons (1–4, absent to highest intensity) and correlated with the level of expression of IFNL1 and IFNB1. (r = correlation coefficient, p<0.0001 for slope deviation from 0). (TIF) [file ppat.1005122.s009.tif]

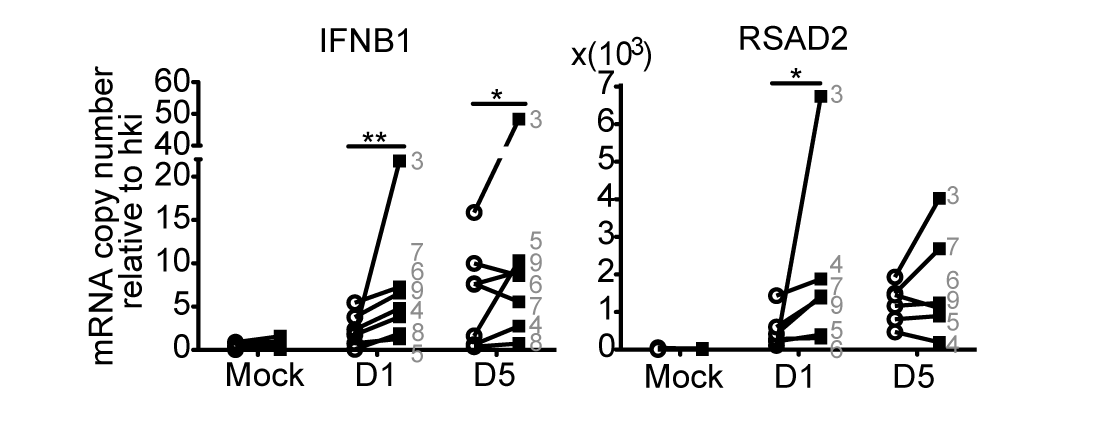

Supplement: S10 Fig — hPCLS were infected with 107 TCID50/slice RSV-LD or RSV-HD and kept in culture for 5 days. Slices from 7 different donors were tested for expression of RSV G or antiviral genes mRNA by qRT-PCR. Lung slices from patient 1 and 2 were used for setting up the system. Gene expression is shown as copy number relative to a house keeping gene expression index determined from ACTB and GAPDH. Results showed paired data, numbers represent the corresponding patient lung slice. (*p<0.05, **p<0.01 by one-tailed Wilcoxon matched-pairs signed rank test). (TIF) [file ppat.1005122.s010.tif]

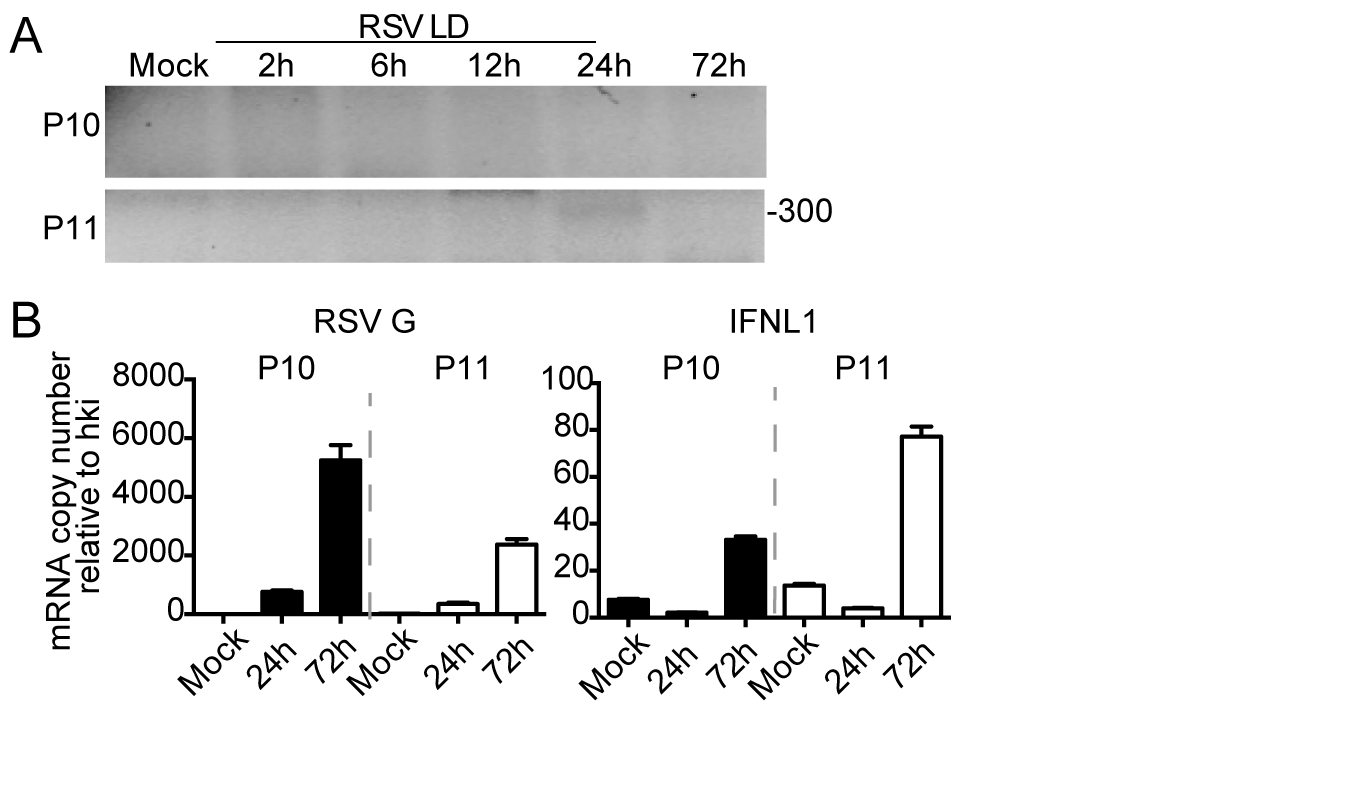

Supplement: S11 Fig — hPCLS were infected with 106 TCID50/slice RSV-LD and kept in culture for up to 3 days. Slices from 2 different donors were tested for DVGs by DI-RT-PCR (A) and expression of RSV G or antiviral genes mRNA by qRT-PCR (B). Error bars indicate mean ± SEM of three slices from the same patient. (TIF) [file ppat.1005122.s011.tif]
