# Supplementary material for: Immunostimulatory Defective Viral Genomes from Respiratory Syncytial Virus Promote a Strong Innate Antiviral Response during Infection in Mice and Humans
Source: PLoS Pathog. 2015 Sep 3;11(9):e1005122. doi: 10.1371/journal.ppat.1005122 (PMC4559413; doi:10.1371/journal.ppat.1005122)
Supplement: S1 Table — Names in uppercase correspond to genes in humans and names in lowercase indicate genes from mice. (DOC) [file ppat.1005122.s012.doc]

**Supporting Information**

**Table S1. RT-qPCR Primer List**

| **Gene** | **Forward** | **Reverse** |
| --- | --- | --- |
| ACTB | 5’AGAGCTACGAGCTGCCTGAC3’ | 5’CGTGGATGCCACAGGACT3’ |
| GAPDH | 5’GCAAATTCCATGGCACCGT’ | 5’TCGCCCCACTTGATTTTGG3’ |
| IFNB1 | 5’GTCAGAGTGGAAATCCTAAG3’ | 5’ACAGCATCTGCTGGTTGAAG3’ |
| IFNA4 | 5’TCTTTACTGATGGCCGTGCT3’ | 5’TCAAGGCCCTCCTATTACCC3’ |
| IFNL1 | 5’CGCCTTGGAAGAGTCACTCA3’ | 5’GAAGCCTCAGGTCCCAATTC3’ |
| RSAD2 | 5’TGCTTTTGCTTAAGGAAGCTG3’ | 5’TCTACTTTGCAGAACCTCACCA3’ |
| IFITI | 5’GGATTCTGTACAATACACTAGAAACCA3’ | 5’CTTTTGGTTACTTTTCCCCTATCC3’ |
| IRF1 | 5’AGGCTACATGCAGGACTTG3’ | 5’ACTGGGATGTGCCAGTCG3’ |
| IRF3 | 5’AGAGGCTCGTGATGGTCAA3’ | 5’TGTGCAGGTCCACAGTATTG3’ |
| *RSV g* | 5’AACATACCTGCCCAGAATC3’ | 5’GGTCTTGACTGTTGTAGATTGCA3’ |
| *Rsp11* | 5’CGTGACGAAGATGAAGATGC3’ | 5’ GCACATTGAATCGCACAGTC3’ |
| *α-tubulin* | 5’ TGCCTTTGTGCACTGGTATG3’ | 5’ CTGGAGCAGTTTGACGACAC3’ |
| *Ifnb1* | 5’AGATGTCCTCAACTGCTCTC3’ | 5’ AGATTCACTACCAGTCCCAG3’ |
| *Ifnl2* | 5’ AGGTCTGGGAGAACATGACTG3' | 5’CTGTGGCCTGAAGCTGTGTA3’ |
| *Ifit1* | 5’CAACCAAGTGTTCCAATGCTCCTTC3’ | 5’TTGCCTGCTAGACAGGGTCAGAAAG3’ |
| *Isg15* | 5’ACGATTTCCTGGTGTCCGTGACTAA3’ | 5’CCAGACCCAGACTGGAAAGGGTAAG3’ |
| *Tnfα* | 5’TCACTGGAGCCTCGAATGTC3’ | 5’GTGAGGAAGGCTGTGCATTG3’ |
| *Il1a* | 5’TTGGTTAAATGACCTGCAACA3’ | 5’GAGCGCTCACGAACAGTTG3’ |
| *Il1b* | 5’CCTCTGATGGGCAACCACTT 3’ | 5’TTCATCCCCCACACGTTGAC 3’ |
| *Il6* | 5’ACAGAAGGAGTGGCTAAGGA 3’ | 5’CGCACTAGGTTTGCCGAGTA3’ |
